# A new hynobiid-like salamander (Amphibia, Urodela) from Inner Mongolia, China, provides a rare case study of developmental features in an Early Cretaceous fossil urodele

**DOI:** 10.7717/peerj.2499

**Published:** 2016-10-05

**Authors:** Jia Jia, Ke-Qin Gao

**Affiliations:** School of Earth and Space Sciences, Peking University, Beijing, China

**Keywords:** Early Cretaceous, Larval–juvenile–adult forms, New fossil taxon, Hynobiid-like salamander, Developmental features

## Abstract

A new fossil salamander, *Nuominerpeton aquilonaris* (gen. et sp. nov.), is named and described based on specimens from the Lower Cretaceous Guanghua Formation of Inner Mongolia, China. The new discovery documents a far northern occurrence of Early Cretaceous salamanders in China, extending the geographic distribution for the Mesozoic fossil record of the group from the Jehol area (40th–45th parallel north) to near the 49th parallel north. The new salamander is characterized by having the orbitosphenoid semicircular in shape; coracoid plate of the scapulocoracoid greatly expanded with a convex ventral and posterior border; ossification of two centralia in carpus and tarsus; and first digit being about half the length of the second digit in both manus and pes. The new salamander appears to be closely related to hynobiids, although this inferred relationship awaits confirmation by research in progress by us on a morphological and molecular combined analysis of cryptobranchoid relationships. Comparison of adult with larval and postmetamorphic juvenile specimens provides insights into developmental patterns of cranial and postcranial skeletons in this fossil species, especially resorption of the palatine and anterior portions of the palatopterygoid in the palate and the coronoid in the mandible during metamorphosis, and postmetamorphic ossification of the mesopodium in both manus and pes. Thus, this study provides a rare case study of developmental features in a Mesozoic salamander.

## Introduction

Hynobiids are a group of small to medium sized salamanders, consisting of 64–66 extant species in 9–11 genera (Fei et al., 2006; Peng et al., 2010; AmphibiaWeb, 2016; Frost, 2016). Hynobiids are commonly known as “Asian salamanders,” as extant members are distributed across Asia with a single species (*Salamandrella keyserlingii*) spreading into European Russia (Zhang et al., 2006; Frost, 2016). Monophyly of Hynobiidae was questioned by some authors (e.g., Trueb & Cloutier, 1991; Trueb, 1993), but is supported by phylogenetic analyses based on molecular data (Weisrock, Harmon & Larson, 2005; Zhang et al., 2006; Peng et al., 2010; Pyron & Wiens, 2011; Weisrock et al., 2013; Chen et al., 2015) and combined morphology/molecular data (Wiens, Bonett & Chippindale, 2005). Hynobiidae have long been regarded as a primitive group because they exhibit a suite of plesiomorphic characters including: external fertilization (Dunn, 1923; Noble, 1931; Regal, 1966); a separate angular in the mandible (Noble, 1931; Hecht & Edwards, 1977); all spinal nerves but the first pair exit intervertebrally (Edwards, 1976); a large number of chromosomes and presence of microchromosomes (Hecht & Edwards, 1977; Sessions, 2008). The origin of hynobiids was hypothesized as “out of north China” (Zhang et al., 2006), and the time of origin recently was hypothesized to be between 120–150 Ma based on an analysis of 29 nuclear genes (Chen et al., 2015).

Hynobiidae are classified in the Cryptobranchoidea, along with Cryptobranchidae (the giant salamanders) as sister clades. With accumulating data from Mesozoic fossil discoveries worldwide in the past decades, the evolutionary history of Cryptobranchoidea can be traced back to the Middle Jurassic Bathonian time (Gao & Shubin, 2003; Gao, Chen & Jia, 2013; Skutschas, 2013; Skutschas, 2016). Cryptobranchidae have a relatively abundant fossil record, with more than 12 fossil species having been reported from Cenozoic strata in Eurasia and North America (Estes, 1981; Vasilyan & Böhme, 2012; Vasilyan et al., 2013 and references therein). *Chunerpeton* represents the earliest fossil cryptobranchid and it is based on a fully articulated specimen from the Middle Jurassic Haifanggou Formation (Gao & Shubin, 2003). The Haifanggou Formation has yielded an ^40^Ar/^39^Ar age of 166.7 ± 1.0 Ma, based on two tuff samples from the lower part of the formation in Beipiao Basin (Chang et al., 2014), western Liaoning Province, China.

In contrast, Hynobiidae have an extremely poor fossil record. The European *Parahynobius* is known by maxillary, vertebral and appendicular material from the upper Miocene of Hungary and lower Pleistocene of Romania (Venczel, 1999a; Venczel, 1999b). The type species *Parahynobius betfianus* is based on a right premaxilla (holotype MTC No. 19913) plus isolated vertebrae and appendicular elements from the lower Pleistocene of Romania (Venczel, 1999a; Venczel, 1999b). The holotype premaxilla has a prominent but wide pars dorsalis (dorsal process), indicating the absence of an anterodorsal fenestra (premaxillary fontanelle). Largely based on this feature, the fossil taxon has been interpreted as a close relative of the extant *Hynobius*-group (Venczel, 1999b). However, several posterior trunk vertebrae referred to the same species have a transverse process with separated diapophysis and parapophysis, a clear indication of having bicapitate ribs, whereas most of other vertebrae have unicapitate transverse processes (Venczel, 1999a; Venczel, 1999b). A second species *Parahynobius kordosi* is based on trunk and caudal vertebrae from the upper Miocene (MN 13) of Hungary, with its holotype as a posterior trunk vertebra (HGM No. V20780) also displays bicapitate transverse process (Venczel, 1999a). Referral of the vertebrae with both bicapitate and unicapitate transverse process to the same taxon is problematic. Because the fossil material consists of isolated bones, there is always a risk of incorrect associating vertebrae of different taxa to a single salamander. To our knowledge, there are no extant hynobiids showing mixture of bicapitate and unicapitate ribs. Articulated material seems necessary to resolve this problem.

Another fossil hynobiid, *Ranodon* cf. *sibiricus* [*Ranodon* cf. *R*. *sibiricus*], is known by eight isolated vertebrae, a left femur and two incomplete right humeri from the upper Pliocene (early Villafrancian in age or equivalent to European MN 16; Tjutkova, 1990) Kiikbai Formation of southern Kazakhstan (Averianov & Tjutkova, 1995).

A more recent discovery in Russia is a single trunk vertebra (ZIN PH1/181) identified as *Salamandrella* sp. from the lower Miocene Khalagay Formation (Syromyatnikova, 2014). Subfossils of *Salamandrella keyserlingii*were reported but remain undescribed from Holocene deposits in Siberia (Khozatski, 1982). So far, there are no known Paleogene fossils for Hynobiidae.

As for the Mesozoic record, *Liaoxitriton zhongjiani* from the Lower Cretaceous of western Liaoning was described as a hynobiid-like salamander (Dong & Wang, 1998), and has been recognized as the earliest fossil hynobiid based on a suite of shared derived features including: the presence of radial loops in the hyobranchium; subarcualis rectus I encasing both the first and second ceratobranchial; transverse and arched vomerine tooth row; and deeply notched posterolateral border of the vomer for choana (Chen & Gao, 2009). In addition, the optic foramen opening at the notched posterior border of the orbitosphenoid is another feature supporting the affiliation of *Liaoxitriton zhongjiani* to Hynobiidae (this study). No isotopic date is available for the fossil beds of *Liaoxitriton*, but the age of the fossil beds has been estimated as 122–125 Ma or Aptian based on stratigraphic correlations with the Yixian Formation (Zhang, Gao & Wang, 2004; Gao, Chen & Jia, 2013).

Another hynobiid-like taxon, *Liaoxitriton daohugouensis* was reported as congeneric with *L. zhongjiani* (Wang, 2004). The former species is known by two specimens (IVPP V13393, V14062) reportedly from the Daohugou locality (Wang, 2004), and hence from the Middle Jurassic Haifanggou Formation (see Gao, Chen & Jia, 2013). However, both its actual occurrences in the fossil beds at Daohugou section and its con-generic status with *Liaoxitriton zhongjiani* need to be confirmed (Gao, Chen & Jia, 2013). Other hynobiid-like salamanders known from Lower Cretaceous beds in China include *Laccotriton orientalis* (Gao, Cheng & Xu, 1998), *Sinerpeton fengshanensis* (Gao & Shubin, 2001), *Regalerpeton weichangensis* (Zhang et al., 2009). All these need to be revised and included in a phylogenetic analysis to resolve their relationships with extant and closely related fossil taxa (work in progress).

**Figure 1:**
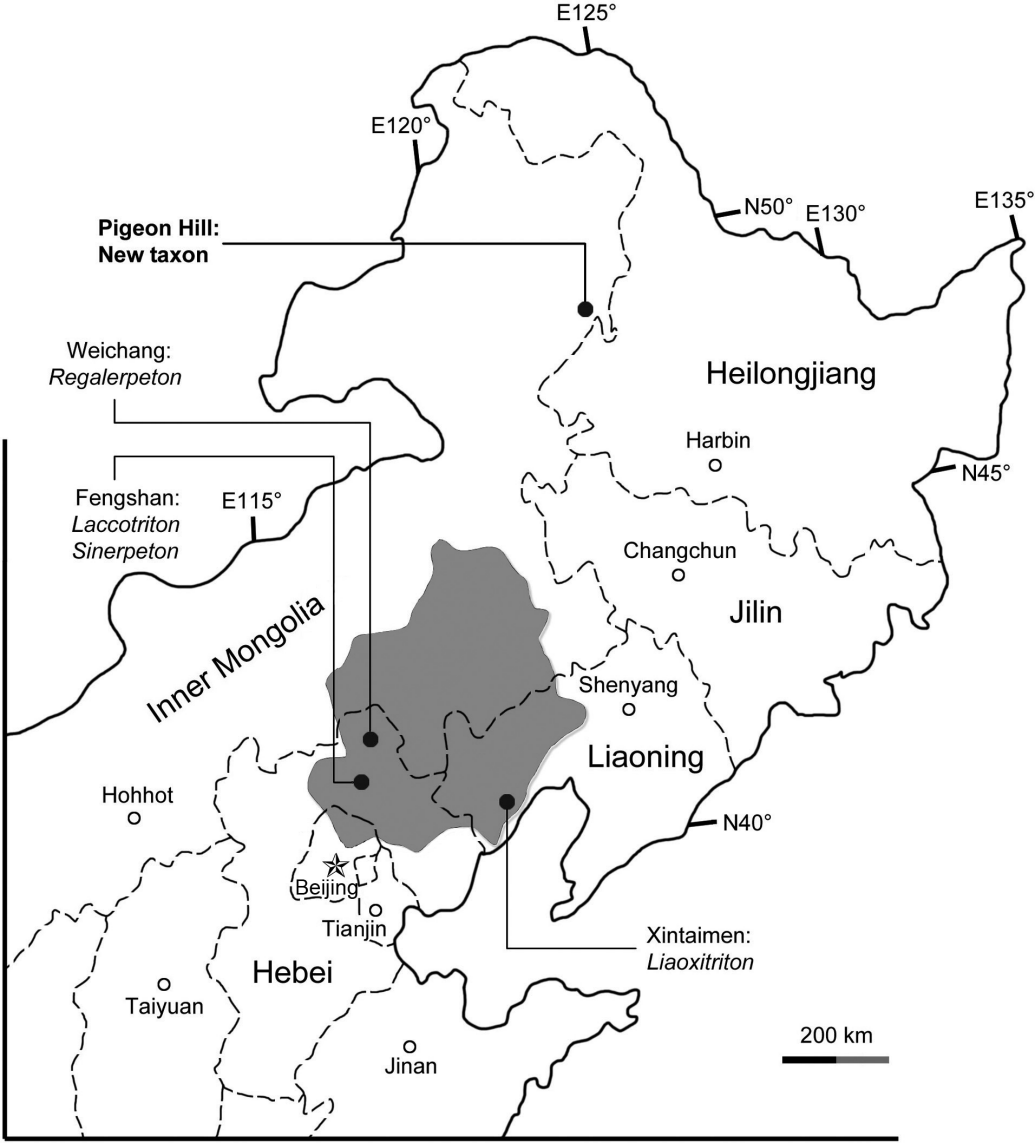
Area map showing geographic distribution of salamander fossil localities in the Lower Cretaceous of northeastern China. The shaded area indicates the former “Jehol Province,” after which the “Jehol Biota” was named.

The purposes of this paper are to name and describe an Early Cretaceous hynobiid-like salamander from eastern Inner Mongolia, China, and to document developmental patterns of cranial and postcranial skeletons for this fossil taxon based on specimens of different developmental stages. We also discuss the higher-level classification of the new salamander, but any consideration of its detailed relationships with extant hynobiids and other hynobiid-like fossil taxa must await the results of another project of ours using combined morphological and molecular data to analyze relationships within Cryptobranchoidea.

The salamander fossils reported in this study were collected from the Pigeon Hill locality (N48°66′43.38″/E123°87′28.8″), near Taipingqiao village, Baoshan Township, Morin Dawa Daur Autonomous Banner of Hulunbuir City, Inner Mongolia, China (Fig. 1). Fossil beds cropping out at the locality pertain to the Lower Cretaceous Guanghua Formation, which consists of a set of volcanic rocks deposited within the Dayangshu Basin, along the south slope of the Greater Khingan Range (Sun et al., 2005; Liu et al., 2008). The Guanghua Formation has been dated at 125 Ma (Heilongjiang Institute of Geological Survey, 2005), and thus is stratigraphically equivalent to the Yixian Formation in western Liaoning Province, which has a geochronological range of 122–129 Ma by Ar^40^/Ar^39^ dating (Chang et al., 2009). Also from the Pigeon Hill locality, well-preserved frog fossils have been recently described and represent a rare fossil record from the far north of China (Gao & Chen, in press). Prior to this discovery, all Early Cretaceous salamander fossils known from China were found within a geographical range limited between 40th–45th parallel in North China from Jehol and nearby areas (Fig. 1; Gao, Chen & Jia, 2013). The new salamander described herein documents a far north (approximately 450 km north of the Jehol area) occurrence of Early Cretaceous salamanders in China, and a significant fossil record of early hynobiid-like salamanders from East Asia.

## Materials and Methods

The new salamander reported here is known from nine two-dimensionally preserved body fossils, collected in the summer of 2013 from the same locality and horizon. The specimens are dorso-ventrally compressed and preserved in pale-grey volcanic shales. The specimens were mechanically prepared using fine needles under a Leica MZ 16 microscope, photographed using a Nikon D90 digital camera and illustrated using Adobe Photoshop CS4. All nine specimens are deposited in the Peking University Paleontological Collections.

Four specimens (PKUP V0417–V0420) with SPL (snout-pelvic length) ranging between 33.9 mm–43.8 mm are identified as larval individuals, because their palatopterygoid is typical for larval salamanders (Rose, 2003). One moderately large specimen (PKUP V0416: SPL of 47 mm) is identified as a postmetamorphic juvenile, as it has the vomer and pterygoid completely re-shaped, but its mesopodium remains unossified. Other large specimens (PKUP V0414, V0415, V0421, V0422) are identified as adults based on their substantially larger size (SPL of 77.7 mm–79.8 mm), extensive limb ossification compared to the juvenile and larval specimens.

Despite their ontogenetic differences, all specimens are referred to the same genus and species, because they all show the diagnostic features of the new taxon (see below). Our morphological description of the new salamander is based mainly on the adult specimens (PKUP V0414, V0415, V0421, V0422), whereas the larval and juvenile specimens provide insights into ontogenetic remodeling of the palate and ossification of the limbs. PKUP V0414 is designated as the holotype, because it is an adult specimen with the best-preserved skull and postcranial skeleton. Anatomical terms used herein follow Francis (1934), Trueb (1993) and Rose (2003) with exceptions explained in the text. SPL and SL (skull length) were measured using calipers.

**Nomenclatural acts**—The electronic version of this article in Portable Document Format (PDF) will represent a published work according to the International Commission on Zoological Nomenclature (ICZN), and hence the new names contained in the electronic version are effectively published under that Code from the electronic edition alone. This published work and the nomenclatural acts it contains have been registered in ZooBank, the online registration system for the ICZN. The ZooBank LSIDs (Life Science Identifiers) can be resolved and the associated information viewed through any standard web browser by appending the LSID to the prefix http://zoobank.org/. The LSID for this publication is: urn: lsid:zoobank.org:pub: B71B1033-62FB-4183-802A-34C431C9CC3D. The online version of this work is archived and available from the following digital repositories: PeerJ, PubMed Central and CLOCKSS.

## Systematic Paleontology

**Table:** 

Class Amphibia Linnaeus, 1758
Subclass Lissamphibia Haeckel, 1866
Superorder Caudata Scopoli, 1777
Order Urodela Duméril, 1806
Suborder Cryptobranchoidea Dunn, 1922
Family Incertae Sedis

Genus *Nuominerpeton* gen. nov.

LSID: zoobank.org:act: 1D7FC3AE-4E45-4FBE-8504-E6C9DA12369E

**Derivation of name**

“Nuomin” for Nuomin River, a tributary of the Nen River running through the Baoshan area east of the fossil locality; “herpeton” (Gr.), a crawling animal.

**Type species**

*Nuominerpeton aquilonaris* sp. nov.

**Diagnosis**

As for the type and only known species.

*Nuominerpeton aquilonaris* sp. nov.

LSID: zoobank.org:act: 2BCD03D7-3D14-47B2-8C20-63FE0F59B958

(Figs. 2–9).

**Figure 2:**
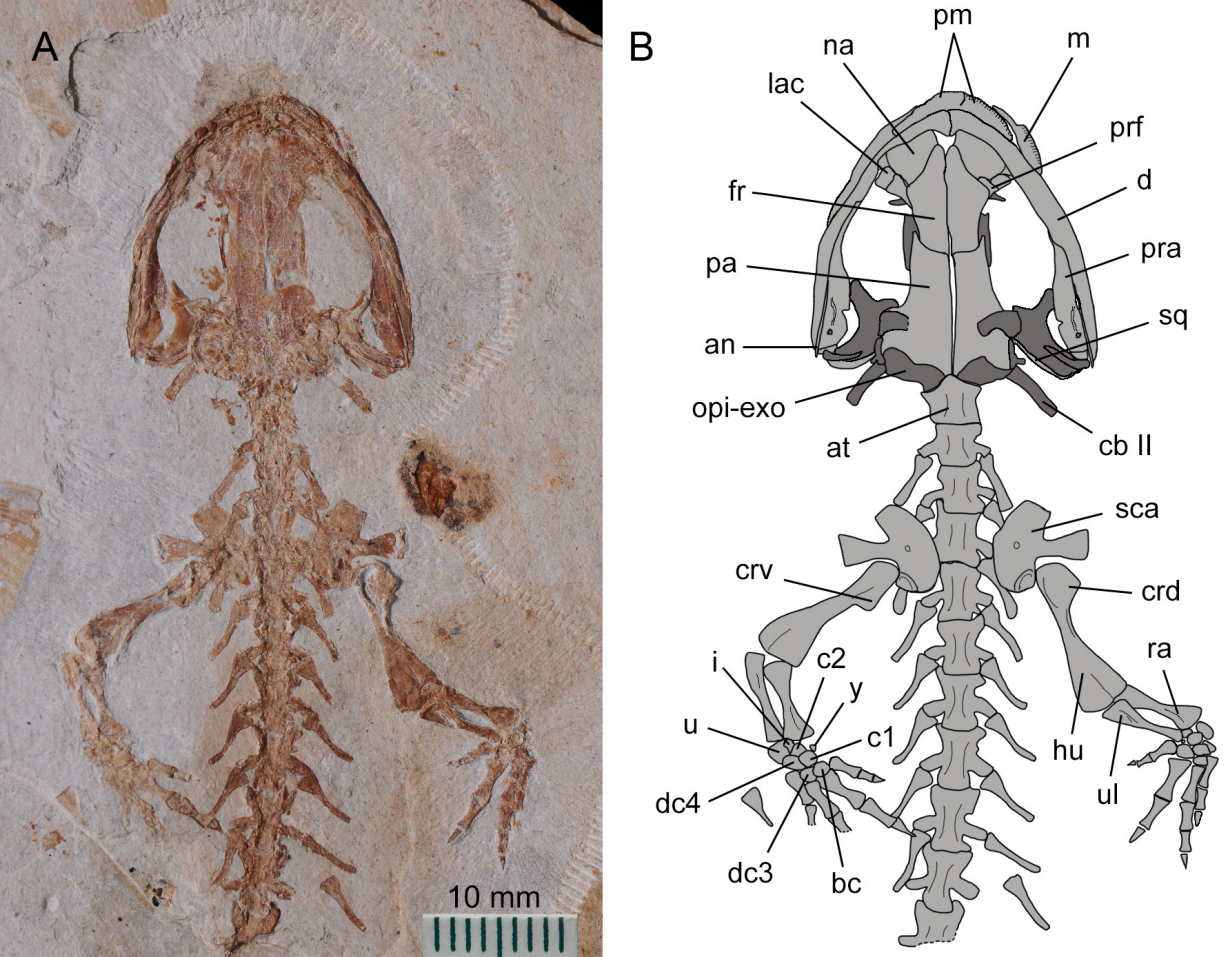
Holotype of *Nuominerpeton aquilonaris* gen. et sp. nov. (part slab of PKUP V0414): photograph (A) and line drawing (B) of the upper body. Note the skull has the roofing elements exposed in ventral view as the consequence of how the shale slabs split through the skeleton. Dark shades denote palatal, braincase and hyobranchial elements.

**Figure 3:**
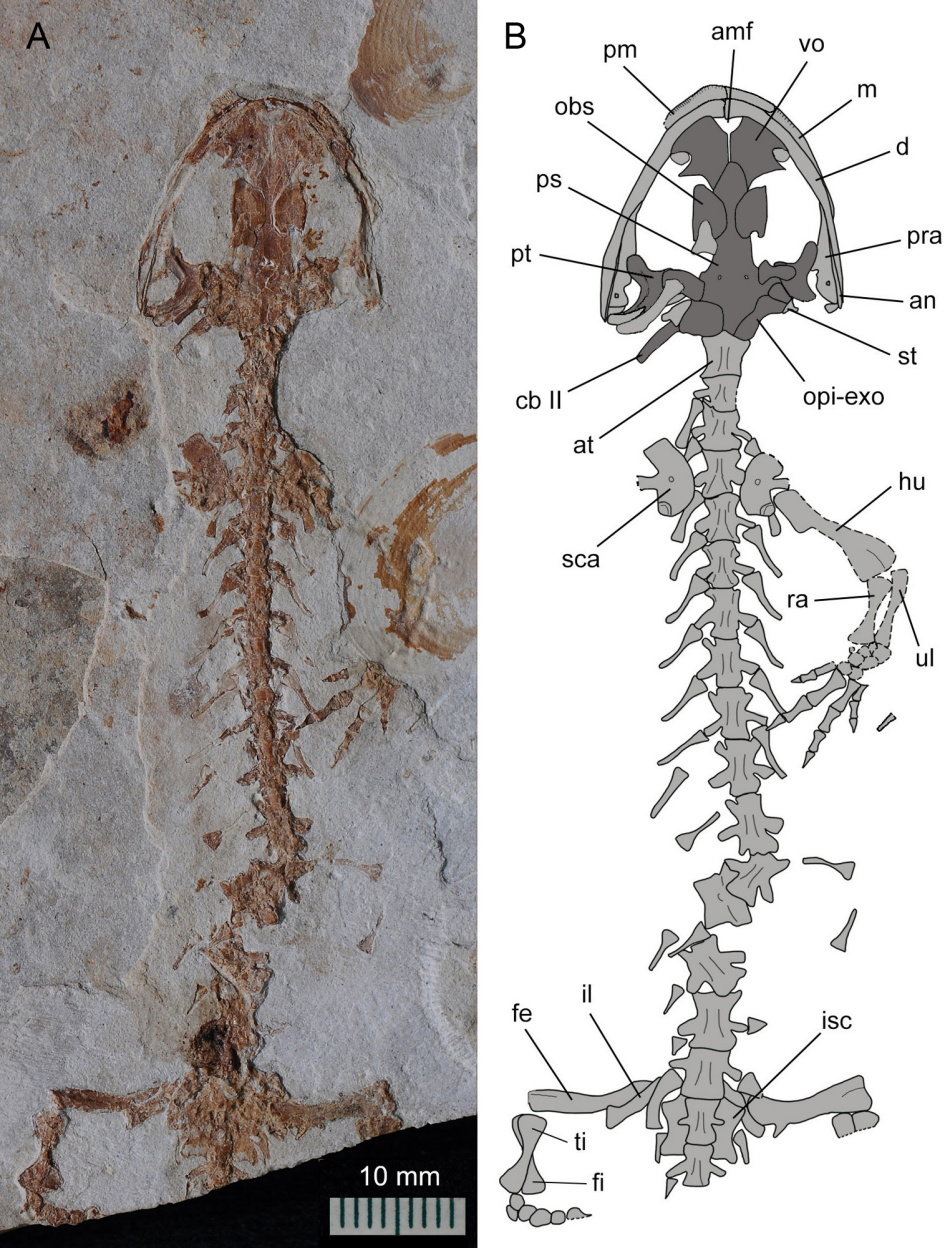
Holotype of *Nuominerpeton aquilonaris* gen. et sp. nov. (counter-part slab of PKUP V0414): photograph (A) and line drawing (B), displaying articulated skeleton with part of the tail missing. Note the skull has the palatal elements exposed in dorsal view resulting from split of shale slabs. Dark shades denote palatal, braincase and hyobranchial elements.

**Derivation of name**

“Aquilonaris” (Latin), northern, referring to the geographic occurrence of the new salamander well north of the Jehol area, where most other Mesozoic salamanders in China are known.

**Holotype**

PKUP V0414, articulated cranial and postcranial skeleton missing distal part of right hind limb and tail, exposed in part and counter-part shale slabs.

**Referred specimens**

PKUP V0415–V0422, all topotypic specimens from the same locality and horizon as the holotype. PKUP V0415, V0421–V0422 are exposed in part and counter-part slabs, whereas other specimens are exposed on a single slab.

**Type locality and horizon**

Pigeon Hill locality (N48°66′43.38″/E123°87′28.8″), near Taipingqiao village, Baoshan Township, Morin Dawa Daur Autonomous Banner of Hulunbuir City, Inner Mongolia, China; Lower Cretaceous Guanghua Formation (Barremian–Aptian), equivalent to the Yixian Formation in western Liaoning.

**Diagnosis**

Medium-sized (maximum known SPL ∼80 mm) metamorphic salamander, differing from other cryptobranchoids in having the following unique suite of characters: lacrimal entering the orbit only, but not the naris; orbitosphenoid semicircular in shape; presacrals 15 in number; caudosacral vertebrae two to three in number; coracoid plate of scapulocoracoid strongly expanded, with straight anterior but convex ventral and posterior borders; humerus with strong projection of dorsal and ventral crests; femur with well-defined, twig-like trochanter; all mesopodial elements but the distal tarsal 5 ossified; ossification of two centralia in both carpus and tarsus; metacarpal II expanded; digit 1 in manus and pes reduced to about half the length of digit 2.

## Description

### General features

The new salamander is medium-sized, with a SPL of 77.7 mm in the holotype (PKUP V0414) and 79.8 mm in the largest adult specimen (PKUP V0421). The skull is slightly longer than wide, with a short and rounded snout as seen in adult specimens (Figs. 2 and 3). The new salamander is a metamorphic form as adult, as evidenced by the lack of internal and external gills, a postmetamorphic type of pterygoid, and an extensively ossified mesopodium in the limb. The tail is slightly longer than the snout-pelvic length, and soft-tissue impressions show that both dorsal and ventral fin folds are absent in the tail.

### Dermal skull roof

The premaxillae are paired, articulating with each other medially to form the anterior margin of the snout. The pars dorsalis (alary process) is a short process overlapping the nasal, as observed in several specimens (PKUP V0416–V0418, V0420, V0422). The tooth-bearing pars dentalis of the premaxilla is preserved and exposed with variable conditions in different specimens. Generally, the pars dentalis is relatively straight in larval forms, but curves and extends perpendicular to the pars dorsalis in adult forms. On the lingual side of the premaxilla, the pars palatina is a narrow ledge that contributes to the anterior portion of the palate.

**Figure 4:**
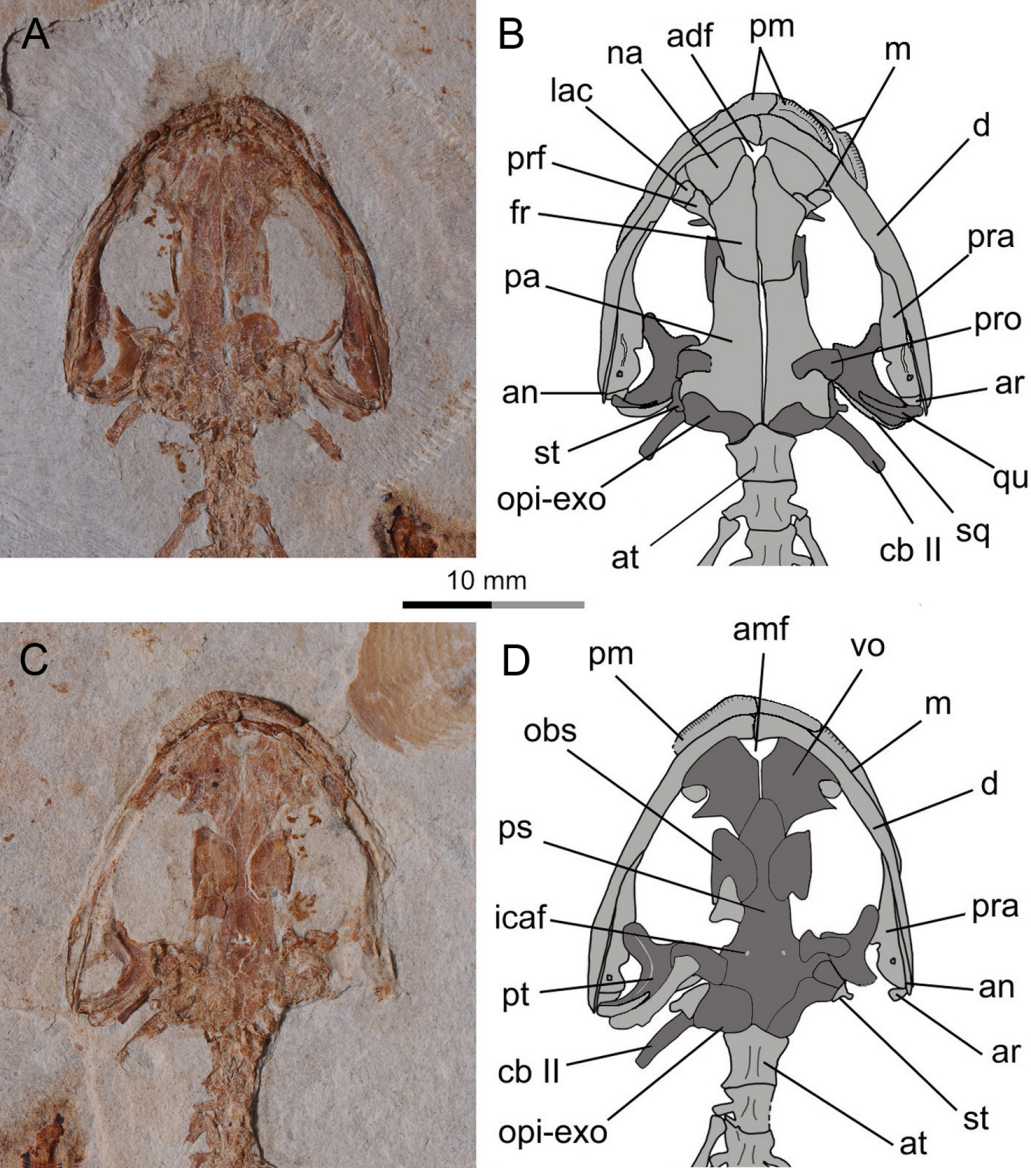
Holotype skull of *Nuominerpeton aquilonaris* gen. et sp. nov. (PKUP V0414): photograph and line drawing of the skull roof (A, B) and palatal (C, D) structures. Note that skull roof elements are exposed in ventral view and the palatal elements in dorsal view as consequence of how the shale slabs split. Dark shades denote palatal, braincase and hyobranchial elements.

The maxilla, as observed in the holotype, is obviously longer than the premaxilla, and has a tapering premaxillary process (anterior process) that makes contact anteriorly with the premaxilla to form the anteroventral border of the external naris. The posterior process of the maxilla approaches the posterior border of the orbit, but does not reach the anterolateral process of the pterygoid. In extant hynobiids, a bony maxilla-pterygoid contact occurs only in *Pachyhynobius* (Fei et al., 2006; Clemen & Greven, 2009). The maxillary pars facialis articulates with the nasal anteromedially, and thus excludes the lacrimal from entering the external naris. Posteromedially, the pars facialis articulates with the lacrimal and is separated from the prefrontal by the latter (Figs. 2 and 4). The pars dentalis carries a row of closely spaced teeth, and the tooth row extends to the posterior extremity of the maxilla.

The paired nasals are laterally expanded and slightly wider than the frontals as a plesiomorphic condition in urodeles (Gao & Shubin, 2012; Jia & Gao, 2016), extant hynobiids also retained such a condition regarding the width of the nasals (Fei et al., 2006). The nasals meet along the midline, except where they are interrupted anteriorly by the anterodorsal fenestra (see below). A midline contact between the nasals occurs in both hynobiids and cryptobranchids, whereas the nasals are separated in salamandroids; the latter pattern is considered as a synapomorphy of salamandroids (Gao & Shubin, 2012). The nasal overlaps the frontal posteriorly, laterally it articulates with the maxilla, and posterolaterally it articulates with both the lacrimal and prefrontal.

The anterodorsal fenestra between the premaxillae and nasals (Figs. 2 and 4) is an opening in the skull roof, corresponding to the anteromedial fenestra in the palate. A clearly defined anterodorsal fenestra is plesiomorphically present in most hynobiids (*Onychodactylus*, *Batrachuperus*, *Liua*, * Pseudohynobius*, *Paradactylodon* and *Ranodon*), but is absent in other genera of the family (*Hynobius*, *Salamandrella*, *Pachyhynobius*) and all cryptobranchids as a derived feature (Sato, 1943; Reilly, 1983; Fei et al., 2006; Zhang et al., 2006; but see Peng et al., 2010). The problematic taxon *Protohynobius* is controversially reported as either having or lacking anterodorsal fenestra by different authors (Fei & Ye, 2000; Peng et al., 2010).

The lacrimal is a small bone at the anterior border of the orbit. As seen in the holotype, the lacrimal articulates with the nasal and prefrontal anteromedially, and with the pars facialis of the maxilla anterolaterally. A lacrimal is plesiomorphically present in the stem caudate *Karaurus* (Ivachnenko, 1978), and also in three families (Hynobiidae, Rhyacotritonidae and Dicamptodontidae) of crown-group urodeles (Trueb, 1993; Rose, 2003). The lacrimal variably enters the naris only (*Batrachuperus*, *Liua tsinpaensis*, *Salamandrella*, and *Dicamptodon*), enters the orbit only (*Pachyhynobius*, *Paradactylodon* and some species of *Hynobius*), or enters both the naris and orbit (*Liua shihi*, *Onychodactylus*, *Protohynobius*, *Pseudohynobius*, *Ranodon*, *Rhyacotriton*, and some species of *Hynobius*) (Duellman & Trueb, 1986; Fei et al., 2006; Clemen & Greven, 2009). However, variable conditions of the lacrimal have been recorded in the literature for *Pachyhynobius shangchengensis*, with entering orbit only (Fei et al., 2006); entering nares only, or entering neither the nares nor orbit (Clemen & Greven, 2009). Whether polymorphism is involved or the differences are observational by different authors need to be scrutinized in future research.

**Figure 5:**
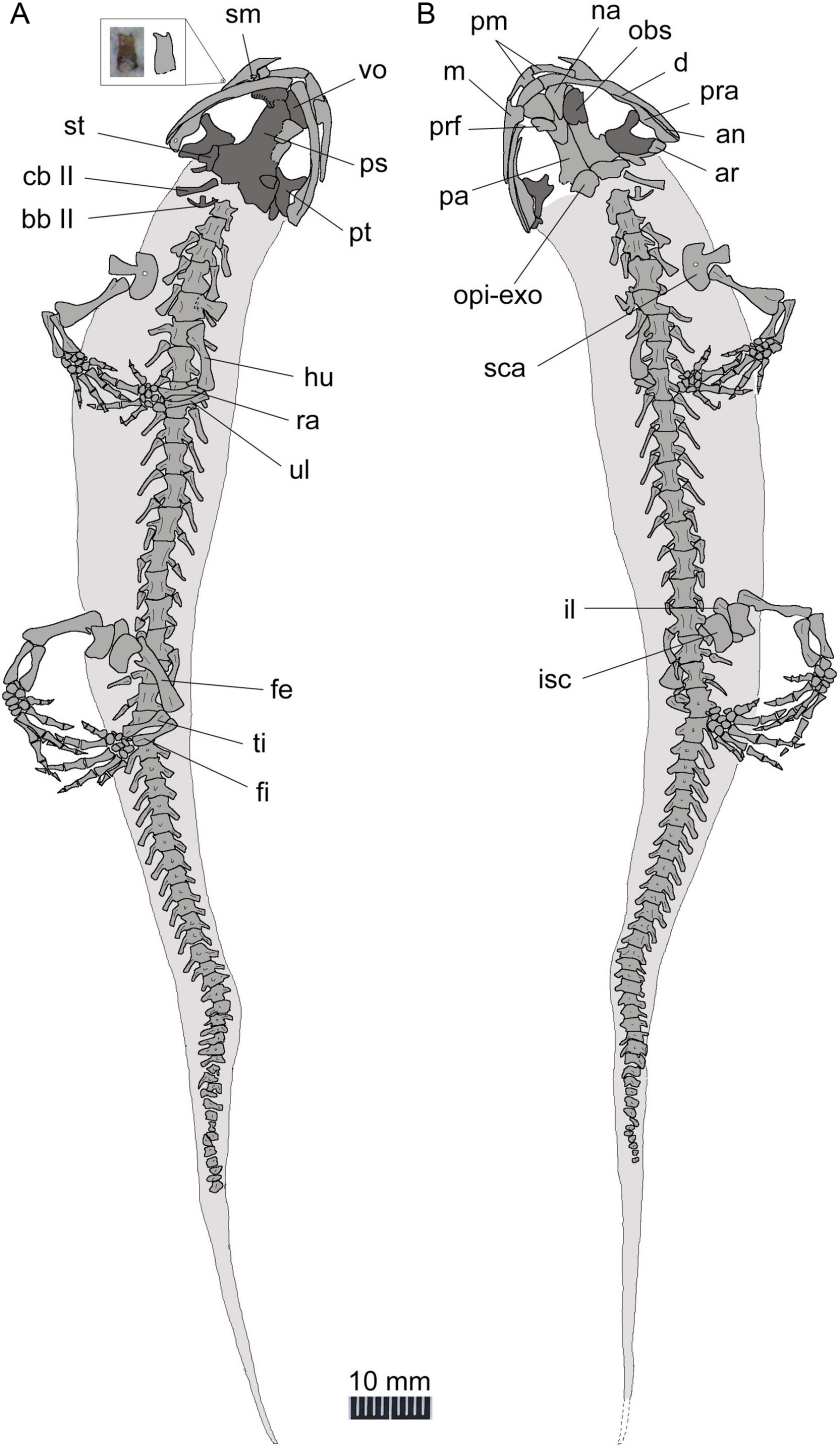
Line drawing of a nearly complete skeleton of *Nuominerpeton aquilonaris* gen. et sp. nov. (PKUP V0421): line drawing of part slab (A) with skeleton exposed in ventral view; line drawing of the counter-part slab (B) with skeleton exposed in dorsal view. Note that the upper left inset is the magnified photograph and line drawing of an isolated maxillary tooth with bicuspid crown. Dark shades denote palatal, braincase and hyobranchial elements.

A septomaxilla is present as in all extant hynobiids and several other urodele families (ambystomatids, dicamptodontids, rhyacotritonids and some plethodontids; Rose, 2003). As observed in the largest specimen (PKUP V0421), the septomaxilla in the new taxon is a small element within the nares. The bone is irregular in shape, being strongly concave at its anterior part and pinched out posteriorly (Fig. 5). The septomaxilla ontogenetically ossifies immediately before or during metamorphosis in the extant hynobiids *Onychodactylus* (Vassilieva, Poyarkov & Iizuka, 2013), *Ranodon* (Schmalhausen, 1968; Lebedkina, 2004), *Salamandrella* (Schmalhausen, 1958; Lebedkina, 1964; Regel, 1970), and *Hynobius* (Vassilieva et al., 2015).

As is typical in extant hynobiids, the prefrontal medially articulates with the nasal and frontal (Fei et al., 2006). In extant cryptobranchids the prefrontal is separated from the nasal by an elongate anterior process of the frontal (Meszoely, 1966). Laterally, the prefrontal borders part of the orbit along with the lacrimal, but is separated from the pars facialis of the maxilla by the latter bone. The absence of a prefrontal-maxilla contact corresponds to the condition of the lacrimal entering the orbit as in many hynobiids (see ‘Description’ above), whereas the presence of prefrontal-maxilla contact corresponds to the condition of the lacrimal entering the naris only in other hynobiids (Meszoely, 1966; Fei et al., 2006).

The frontals are paired with a straight midline suture between them (Fig. 4). The frontal articulates with the nasal and prefrontal anterolaterally, and overlaps the parietal posteriorly. A frontal-maxilla contact is absent. Such a contact occurs only in cryptobranchids, but is absent in all hynobiids.

The parietals are paired, with a straight suture between them along the midline. The anterolateral process of the parietal is extremely short and lacks a contact with the prefrontal as seen in all extant hynobiids (Sato, 1943; Fei et al., 2006). In contrast, a prefrontal-parietal contact is consistently found in extant cryptobranchids (Reese, 1906; Meszoely, 1966). The posterolateral process of the parietal is boot-like, in articulation with the prootic, the opisthotic-exoccipital complex posteroventrally, and with the squamosal laterally. A parietal-squamosal contact is plesiomorphically present in cryptobranchids, most hynobiids except *Onychodactylus* (Smirnov & Vassilieva, 2002a; Fei et al., 2006; Vassilieva, Poyarkov & Iizuka, 2013) and a few species of *Hynobius* (Sato, 1943; Nambu, 1991; Fei et al., 2006).

### Suspensorium

The squamosal is not fully exposed in the holotype, but can be better observed in the juvenile and the three of larval specimens (PKUPV0416–V0419). The squamosal is essentially a transverse bar with an expanded proximal end and a blunt distal end. Its proximal end is in contact with the posterolateral process of the parietal, thus in life dorsally roofing the otic capsule. The distal end overlaps the quadrate and the posterolateral process of the pterygoid as part of the cranio-mandibular suspensorium.

The quadrate is transversely oriented, and makes contact with the ventral side of the squamosal. As observed in the holotype, the quadrate has a tapering medial end but a slightly widened lateral end (Figs. 2 and 4). The quadrate is ventrally concave, displaying a trough-like structure. A quadrate foramen is observed in one juvenile specimen (PKUP V0416), but not in other specimens because in those the lateral end of the element is not exposed. An ossified quadrate can be identified in all four larval specimens (PKUP V0417–V0420), indicating its ossification before metamorphosis. In extant hynobiids, the quadrate ossifies immediately before metamorphosis (Suzuki, 1932: *Onychodactylus*; Lebedkina, 2004: *Salamandrella*), during metamorphosis (Lebedkina, 1964: *Ranodon sibiricus*), or after metamorphosis (Vassilieva et al., 2015: *Hynobius formosanus*).

**Figure 6:**
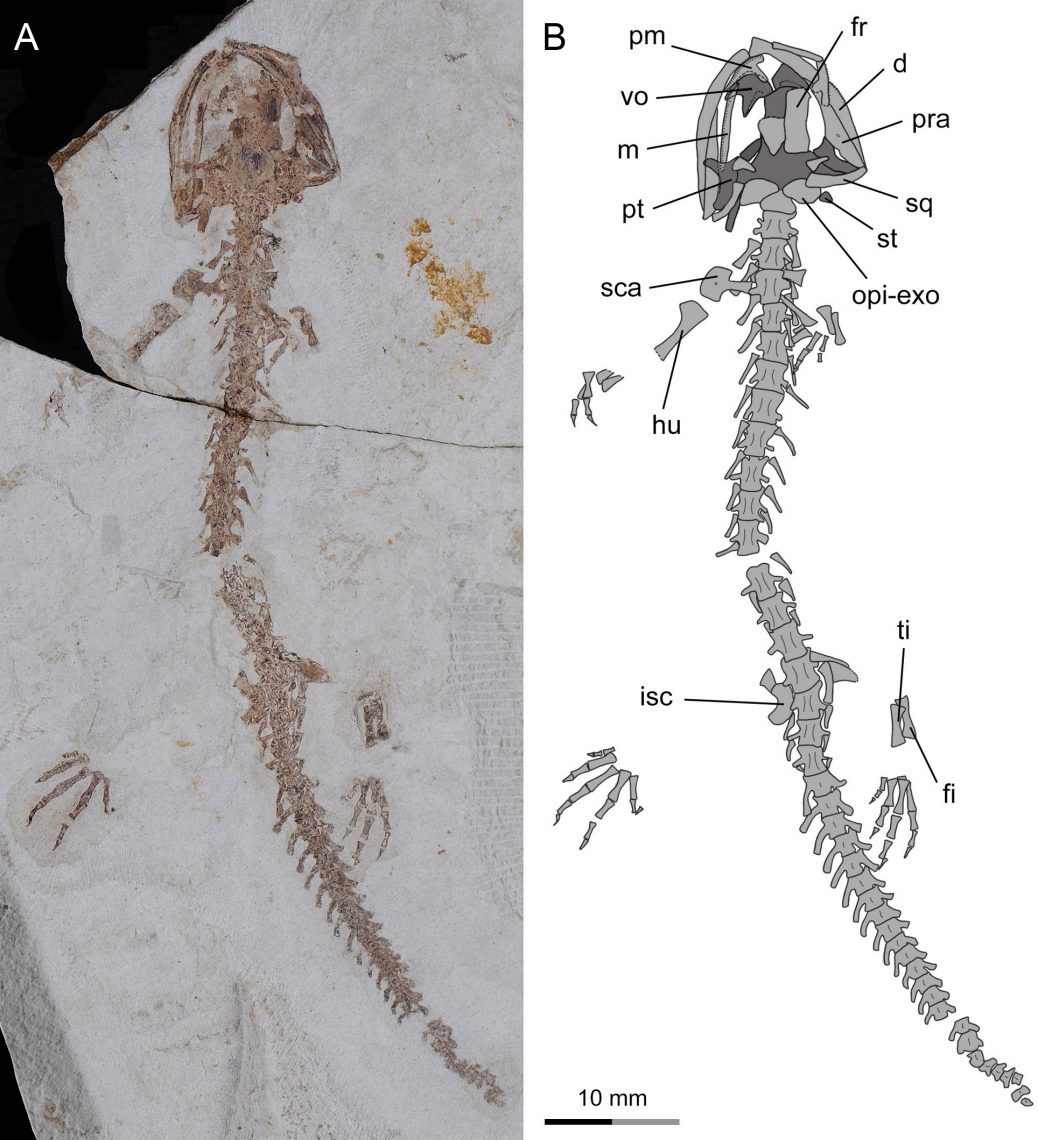
Incomplete skeleton of the only known postmetamorphic juvenile specimen of *Nuominerpeton aquilonaris* gen. et sp. nov. (PKUP V0416): photograph (A) and line drawing (B) of an articulated skeleton exposed in dorsal view. Note that the pterygoid has been re-shaped to the postmetamorphic configuration, but the carpus and tarsus remain unossified. Dark shades denote palatal, braincase and hyobranchial elements.

The pterygoid is triradiate, with a short and blunt palatal process directed anterolaterally. This shape and orientation are seen in the adult (PKUP V0414, V0415, V0421, V0422) and postmetamorphic juvenile (PKUP V0416; Fig. 6) specimens, whereas larval specimens display a different morphology (see below). In adult stages, the pterygoid has a smooth ventral surface, but dorsally the palatal process bears a bony ridge that curves posterolaterally extending along the quadrate process of the bone. That ridge is probably for attachment of the M. levator mandibulae posterior as in the extant salamandrid *Salamandra* (Francis, 1934). The medial process of the pterygoid is short and articulates with the lateral ala of the parasphenoid. The quadrate process is much wider than the palatal process, and is distally in contact with the quadrate and the squamosal.

**Figure 7:**
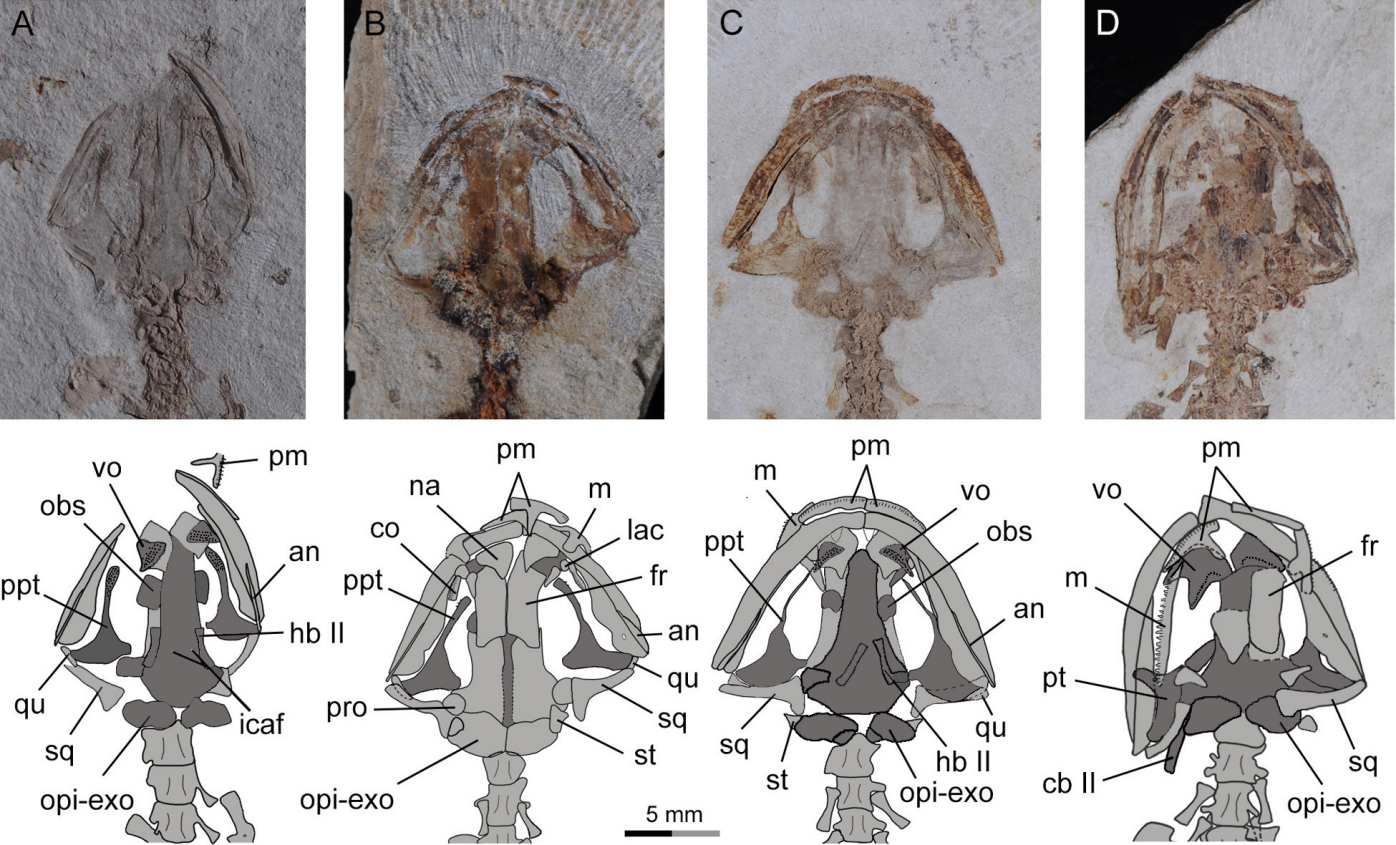
Photographs and line drawings of skull and mandibles in larval and juvenile specimens of *Nuominerpeton aquilonaris* gen. et sp. nov., showing ontogenetic resorption of palatine and anterior pterygoid portions of the palatopterygoid. (A) PKUP V0419 in palatal view, displaying a slightly expanded, dentate palatine portion as part of the palatopterygoid; (B) PKUP V0418 in dorsal view, displaying slightly resorbed palatine as a narrow strip; (C) PKUP V0417 in palatal view, displaying further resorption of the palatopterygoid as metamorphosis approaches; (D) PKUP V0416, a postmetamorphic juvenile specimen in dorsal view, displaying ossified ceratobranchial II and re-shaped pterygoid with palatal process oriented anterolaterally. Dark shades denote palatal, braincase and hyobranchial elements.

As seen in extant salamanders, the four larval specimens display a palatopterygoid with an elongate anterior process in connection with a dentate palatine portion. The anterior process is curved anteromedially (Figs. 7A–7C), sharply different from the anterolateral orientation in juveniles and adults (Figs. 4–6 and 7D). Developmental remodeling of the palatopterygoid, along with re-orientation of the palatal process of the pterygoid, is achieved at metamorphosis (see discussion below).

### Palate

The partes palatina of both premaxilla and maxilla form the anterior part of the palate by articulating with the vomer. No independent palatine is found in any specimens, but a palatine portion of the palatopterygoid does occur in larval specimens (PKUP V0417–V0420; see discussion below) as a common developmental pattern known from other salamanders (e.g., Rose, 2003; Lebedkina, 2004). Also in the palate, an “anteromedial fenestra” (sensu Trueb, 1993) is present, bordered by the premaxillae and vomers (Figs. 3 and 4). This fenestra persists from larval through to the adult stage in this Early Cretaceous salamander. The same fenestra also is known as “space for glands” (Goodrich, 1930) or “internasal space” (Francis, 1934). Such a fenestra is present in all hynobiids at the adult stage, but it is uncertain for *Protohynobius* (see Fei et al., 2006; Xiong et al., 2011). The fenestra is completely closed in extant cryptobranchids as a derived condition within Cryptobranchoidea (Sato, 1943; Elwood & Cundall, 1994; Fei et al., 2006).

The vomer is a broad bony plate, irregular in shape. At the adult stage, the paired vomers meet along the midline for most of their extent, but are notched anteromedially for the anteromedial fenestra. The posterolateral margin of the vomerine plate is deeply notched for the choana, with a short triangular postchoanal process (“retrochoanal process” of Rose, 2003; “preorbital process” of Wake, 1966) directing posterolaterally (Figs. 3 and 4). The posterior process of the vomer is roughly the same in shape and length as the postchoanal process, but is directed posteriorly with its medial edge broadly in contact with the parasphenoid.

Vomerine teeth are not exposed in the holotype, but can be observed in five other specimens (PKUP V0416, V0417, V0419, V0421, V0422). All available specimens have the vomerine teeth preserved as impressions; thus, the actual tooth structure including the crown pattern remains unknown. Larval specimens (PKUP V0417, V0419) display no clearly defined choanal notch, but short and multiple rows of vomerine teeth gathered to form a tooth patch, with the two patches widely separated medially, arranged in a slightly oblique position (Figs. 7A and 7C). In contrast, the postmetamorphic juvenile (PKUP V0416) and adult (PKUP V0421, V0422) specimens display a clearly defined choanal notch, with a single tooth row in transverse position, closely associated with the choana, and evidently arched anteriorly. Those differences show how the vomer and vomerine tooth row have been re-shaped at metamorphosis, as a typical developmental pattern as in extant salamanders (Rose, 2003). A short and arched vomerine tooth row is plesiomorphically present in most hynobiids, including *Batrachuperus*, *Liua*, *Onychodactylus*, *Pachyhynobius*, *Paradactylodon*, *Pseudohynobius*, *Protohynobius*, and *Ranodon* (Fei et al., 2006; Zhang et al., 2006), whereas an elongated vomerine tooth row extending posteriorly far beyond the choana is found as a derived feature in *Hynobius* and *Salamandrella* (Sato, 1943; Fei et al., 2006).

The parasphenoid is a large, azygous bony plate contributing to the palate anteriorly and the braincase floor posteriorly. The cultriform process is broad for most of its length, but has a triangular anterior process wedging between the vomers and terminates anteriorly at the level of the choanae (Figs. 7A and 7C). The posterolateral ala contacts with the medial process of the pterygoid. Medial to the parasphenoid-pterygoid articulation, the parasphenoid is obliquely penetrated by a pair of internal carotid foramina for passage of the internal carotid arteries (Francis, 1934).

### Braincase

As mentioned above, the parasphenoid in the new salamander is widened posteriorly to floor the braincase, and the floor is penetrated by a pair of internal carotid foramina at the base of its lateral alae. The orbitosphenoid (sphenethmoid) part of the lateral wall of the braincase is ossified as a plate, roughly semi-circular in shape as seen in the holotype (PKUP V0414). It has a straight ventral edge for articulation with the parasphenoid, but bows dorsally to form a rounded border for articulation with the frontal and parietal. Because the holotype represents an adult with a well-ossified mesopodium, the semi-circular shape of the orbitosphenoid cannot be logically interpreted as possibly an ontogenetic feature. Instead, we regard that shape as a unique feature diagnostic of the new taxon. In the new salamander, the posterior margin of the orbitosphenoid is notched for the passage of the optic nerve and its associated vessels (Fox, 1959). The large fissure between the orbitosphenoid and prootic probably also served as the passage for the oculomotor nerve as commonly seen in extant hynobiids (Fox, 1959; Rose, 2003).

The prootic is irregular in shape, preserved on the ventral side of the posterolateral process of the parietal. In life, the prootic borders the fenestra ovalis, which is covered by the footplate of the stapes (see below).

The opisthotic is fused with the exoccipital to form the opisthotic-exoccipital complex. This complex borders the fenestra ovalis anteriorly, where it articulates with the footplate of the stapes, and posteriorly it bears an occipital condyle for articulation with the atlas (Figs. 2 and 4). The exoccipital and opisthotic were reported as unfused in the stem caudate *Kokartus* (Skutschas & Martin, 2011). In extant salamanders, the fused opisthotic-exoccipital complex with a free prootic is found in all cryptobranchoids (Carroll & Holmes, 1980; Gao & Shubin, 2012), except in *Onychodactylus*, in which the prootic also is fused with the opisthotic-exoccipital complex (Smirnov & Vassilieva, 2002a; AmphibiaTree, 2007a; Vassilieva, Poyarkov & Iizuka, 2013).

The stapes (columella) has a footplate fused with a short stylus as seen in the holotype specimen (Fig. 4). The stylus has a blunt distal tip, which in life may have a ligament connection with the quadrate and squamosal as known from extant hynobiids (Fox, 1959). None of the available specimens have the stapes preserved well enough to determine whether a stapedial foramen is present. No operculum is found in any specimens of the new salamander. In extant cryptobranchoids, the operculum is absent in all taxa (Elwood & Cundall, 1994; Fei et al., 2006). However, Zhang (1985) identified an operculum in the extant hynobiid *Liua shihi*, but ambiguously described the operculum as fused with the footplate of the stapes. Thus, whether *Liua shihi* has an operculum or not demands further investigation.

### Mandible

The lower jaw consists of the dentary, prearticular, angular and articular in adults. The mentomeckelian fuses with the dentary at the mandibular symphysis, and bears a small posterior mental process as seen in the holotype. A small coronoid with a single tooth row is recognizable in larval specimens (PKUP V0418, V0420; Figs. 7B and 9), but this element is resorbed in all postmetamorphic specimens. Contrary no ossified articular can be found in larval and juvenile forms, but adults have it (see below).

The dentary covers a large part of the lateral aspect of the lower jaw. The anterior end of the dentary is slightly thickened, resulting from fusion with the mentomeckelian. The dentary terminates posteriorly at the level of the cranio-mandibular joint, where it is separated from the prearticular by the splint-shaped angular. Dentary teeth are only poorly preserved in all available specimens, but presumably were pedicellate and bicuspid, identical to the teeth known from the maxilla (see below).

The prearticular is a large element covering most of the medial aspect of the mandible. The prearticular tapers anteriorly, but fails to reach the jaw symphysis. The posterior part of the prearticular arises dorsally as a coronoid process for insertion of mandibular adductor muscles. Below the coronoid process, a small prearticular foramen (“inferior dental foramen” of Francis, 1934) penetrates the prearticular for the passage of the inferior alveolar ramus of the facial nerve (CN VII) and the alveolar artery as seen in the extant salamandrid *Salamandra* (Francis, 1934).

The angular is exposed posteroventrally as an extremely slender splint between the prearticular and dentary. The splint pinches out anteriorly at the same level as the anterior tip of the pterygoid. Close to the anterior end of the splint, a small angular foramen opens at the suture between the angular and the prearticular (Fig. 4). Such a foramen serves for the passage of the ramus mandibularis of the Cranial Nerve V in the plethodontid *Pseudotriton* (Joubert, 1961).

The articular is a tiny bone ossified posterodorsally at the end of the lower jaw as observed in adult specimens. The absence of the bone in larval (PKUP V0417–V0420) and postmetamorphic juvenile (PKUP V0416) specimens indicates its delayed ossification after metamorphosis as occurs in extant hynobiids (Smirnov & Vassilieva, 2002a; Smirnov & Vassilieva, 2002b; Lebedkina, 2004; Vassilieva, Poyarkov & Iizuka, 2013; Vassilieva et al., 2015). Among extant cryptobranchoids, the articular is absent by fusion with the prearticular in Cryptobranchidae but is present in Hynobiidae (Zhang, 1985; Rose, 2003; Clemen & Greven, 2009; Zhang, Liu & Zhao, 2009; Vassilieva et al., 2015). Ontogenetically, the articular, if present, ossifies at a later stage than the septomaxilla as summarized by Rose (2003: Table 1); therefore, the presence of an ossified articular as in our four large specimens can be used as a reliable indicator of maturity in a fossil form.

### Dentition

Both marginal and vomerine teeth are poorly preserved in all available specimens. However, isolated maxillary teeth display pedicely, with bicuspid crown patterns, as observed in the largest adult specimen PKUP V0421. In this specimen, most teeth were shattered when the shale slabs were split, but one tooth that detached from the left maxilla before fossilization of the specimen left a clear impression displaying a bicuspid crown pattern (Fig. 5). In addition, bicuspid crowns on maxillary teeth are also observed in the postmetamorphic juvenile specimen (PKUP V0416).

In contrast, a small larval specimen (PKUP V0420) has the paired premaxillae exposed in medial view, showing that each element carries about 20 teeth. The teeth have a pointed tip, indicating the presence of monocuspid crowns. The premaxillary teeth in the latter specimen are apparently pedicellate, indicating that in this fossil form the pedicellate condition was achieved earlier than bicuspid crown pattern before metamorphosis. Such a developmental pattern has been documented for extant salamanders, including the hynobiids *Hynobius nebulosus* and *Salamandrella keyserlingii* (Greven & Clemen, 1985), *Hynobius formosanus* (Vassilieva et al., 2015), *Onychodactylus fischeri* (Smirnov & Vassilieva, 2002a), *Onychodactylus japonicus* (Vassilieva, Poyarkov & Iizuka, 2013), and *Ranodon sibiricus* (Vassilieva & Smirnov, 2001).

### Hyobranchial apparatus

As a consequence of how the shale slabs split, the counter-part slab of the holotype exposes the dorsal aspect of the palate; hence, the hyobranchial apparatus is largely obscured by the parasphenoid. However, the well-ossified ceratobranchial II is clearly visible, due to its posterolateral position in relation to the braincase floor (Fig. 4). Several other specimens (e.g., PKUP V0417, V0419, V0421, V0422) provide information on the ossification of hyobranchial elements, including basibranchial II, hypobranchial II and ceratobranchial II.

The basibranchial I is cartilaginous in all hynobiids, except in *Onychodactylus*, in which the element partly ossified as a short stub (Smirnov & Vassilieva, 2002a; Xiong et al., 2013a). Basibranchial II is ossified with variable contours in all hynobiids, except *Onychodactylus*, in which the element is completely lacking (Xiong et al., 2013a). Interestingly, Smirnov & Vassilieva (2002a) reported that two specimens of *Onychodactylus fischeri* display possible atavistic ossification of a tiny basibranchial II. As in most hynobiids, no trace of ossification of the basibranchial I is seen in the new salamander, whereas the basibranchial II is ossified as an anchor-shaped structure in adults, with a robust median stem and much slender lateral branches curved anterolaterally (Fig. 5; PKUP V0421, V0422). A recent study (Jia & Gao, 2016) recognized the anchor-shaped basibranchial II as a plesiomorphic feature in Urodela by comparison with the stem caudate *Karaurus* and fossil and extant urodeles.

All specimens exposing the skull in palatal view display no ossification of hypobranchial I or ceratobranchial I. This pattern corresponds to that in all hynobiids, except *Pachyhynobius*, which has hypobranchial I ossified but ceratobranchial I remains cartilaginous (Fei et al., 2006; Clemen & Greven, 2009; Xiong et al., 2013a). In the new salamander, the postmetamorphic juvenile (PKUP V0416) and all adult specimens (PKUP V0414, V0415, V0421, V0422) display ossification of both hypobranchial II and ceratobranchial II as separate elements, whereas the two larval specimens in which the relevant area is exposed (PKUP V0417, V0419) show only ossified hypobranchial II. This size-related difference indicates that ossification of the ceratobranchial II is closely associated with the life-history transition of the salamander at metamorphosis as seen in extant hynobiids (Ma, 1964; Smirnov & Vassilieva, 2002a; Vassilieva, Poyarkov & Iizuka, 2013; see discussion below).

### Axial skeleton

The axial skeleton of the new salamander consists of an atlas, 14 trunk vertebrae, one sacral, two to three caudosacrals and a maximum 36 caudals. Information on the neural arch and spinal nerve foramina cannot be obtained because those regions are damaged or not exposed as a consequence of how the shale slabs split. The centrum, as observed in PKUP V0414 and V0415, can be determined as amphicoelous, a general pattern for most salamanders other than the opisthocoelous condition in salamandrids, plethodontids and extinct batrachosauroidids (Wake & Lawson, 1973; Estes, 1981).

The atlas is hourglass-shaped, and is slightly wider than the following presacrals. The odontoid process (tuberculum interglenoideum) is subtriangular in shape in ventral view (PKUP V0414, V0421). Dorsolateral to the odontoid process are the paired cotyles for articulation with the occipital condyles. No transverse processes or free ribs are associated with the atlas as is commonly seen in most extant salamanders. A pair of rudimentary transverse processes is only found in the atlas of some but not all neotenic forms (Skutschas & Krasnolutskii, 2011; Jia & Gao, 2016).

**Figure 8:**
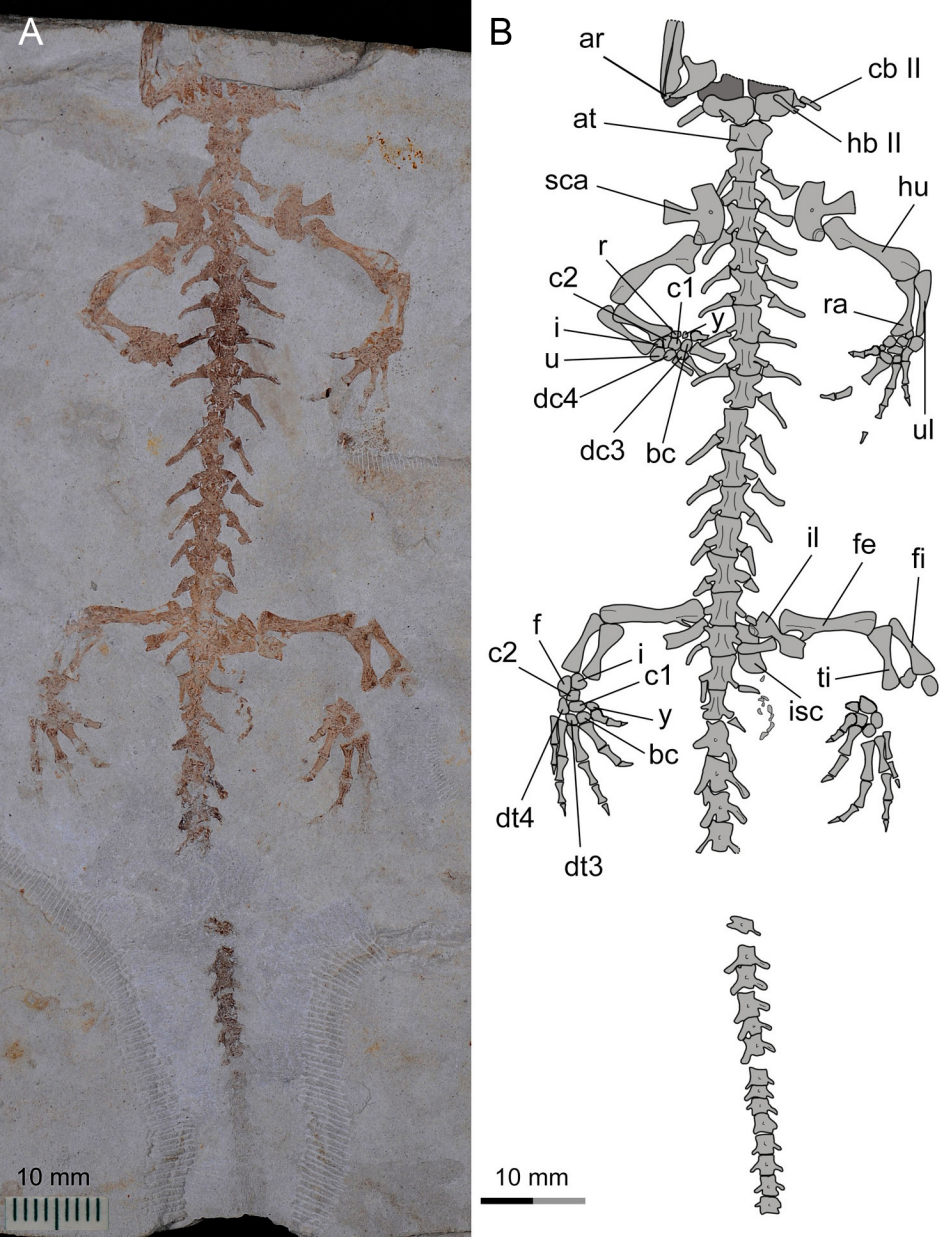
Referred specimen of *Nuominerpeton aquilonaris* gen. et sp. nov. (PKUP V0415): photograph (A) and line drawing (B) of the incomplete skeleton in ventral view.

Trunk vertebrae slightly increase in length posteriorly until the 10th vertebra, with the more posterior being apparently similar in length and slightly shorter than the 10th (Figs. 3, 5 and 8). All trunk vertebrae have a pair of short transverse processes laterally in articulation with unicapitate ribs. The possession of unicapitate ribs is a salient feature indicating affiliation of the new salamander to the suborder Cryptobranchoidea, rather than to the Salamandroidea (Duellman & Trueb, 1986; Gao & Shubin, 2012). The first three pairs of trunk ribs are more robust than the others, with a slightly expanded distal end for insertion of pectoral muscles. The ribs following the anteriormost three pairs are roughly the same length, but those associated with the last four to five trunk vertebrae are noticeably reduced posteriorly, with the last pair being merely a short stub.

The sacral vertebra is roughly the same size as the last trunk vertebra, but the sacral rib is elongate and about the same length as the centrum. The distal end of the sacral rib is slightly expanded, probably for the attachment of the ligament connecting that rib to the ilium in life (Duellman & Trueb, 1986). The two or three rib-bearing vertebrae immediately following the sacral vertebra are identified as caudosacrals as defined by Worthington & Wake (1972). Three specimens (PKUP V0414, V0415, V0422) have two caudosacrals, whereas the other specimens have three. Similar individual variation in the number of caudosacrals is also commonly seen in extant hynobiid species (Xiong et al., 2013b). The free ribs associated with the caudosacrals are slender, short and oriented posterolaterally.

Vertebrae of the caudal series following the caudosacrals are missing in the holotype, but are preserved in other adults (PKUP V0415, V0421, V0422), the juvenile (PKUP V0416) and one of the larval specimens (PKUP V0420). All caudal vertebrae in these specimens have been rotated at a 90° angle in relation to the horizontal body plane, and thus have the neural and haemal arches exposed in lateral view (Figs. 5 and 8). The neural spine is short and directed posterodorsally. The haemal spine of the first caudal bends posteriorly at a much greater angle than those of the remaining caudals, the spine of which slants uniformly at the right angle to the long axis of the centrum. The total number of caudal vertebrae varies in keeping with size and presumably age differences of specimens. The largest specimen in the collection (PKUP V0421) has 36 caudals for the complete tail, whereas the larval specimen (PKUP V0420) has 28 caudals. Other specimens have part of the tail missing; therefore, actual counts for total number of caudal vertebrae cannot be obtained for these specimens.

### Appendicular skeleton

The ossified pectoral girdle consists of a single scapulocoracoid as commonly seen in all salamanders, except sirenids, which are unique among salamanders in having a separate scapular and coracoid (Noble, 1931). The scapular portion is a trapezoidal blade, expanded dorsally, but constricted ventrally and fused with the coracoid plate. The scapulocoracoid in *Nuominerpeton* is notable for having a strongly expanded coracoid plate, the anteroposterior dimension of which is almost twice the length of the scapular portion. The coracoid plate has a straight anterior border, but both the ventral and posterior borders are convex. The supracoracoid foramen (coracoid foramen) penetrates the coracoid plate directly below the scapular blade (Figs. 2, 3, 5 and 8), where it serves for the passage of the supracoracoideus nerve and the associated artery and vein (Francis, 1934). The glenoid fossa is close to the posterodorsal border of the coracoid plate, where it forms an ellipsoid concavity for articulation with the humerus.

**Figure 9:**
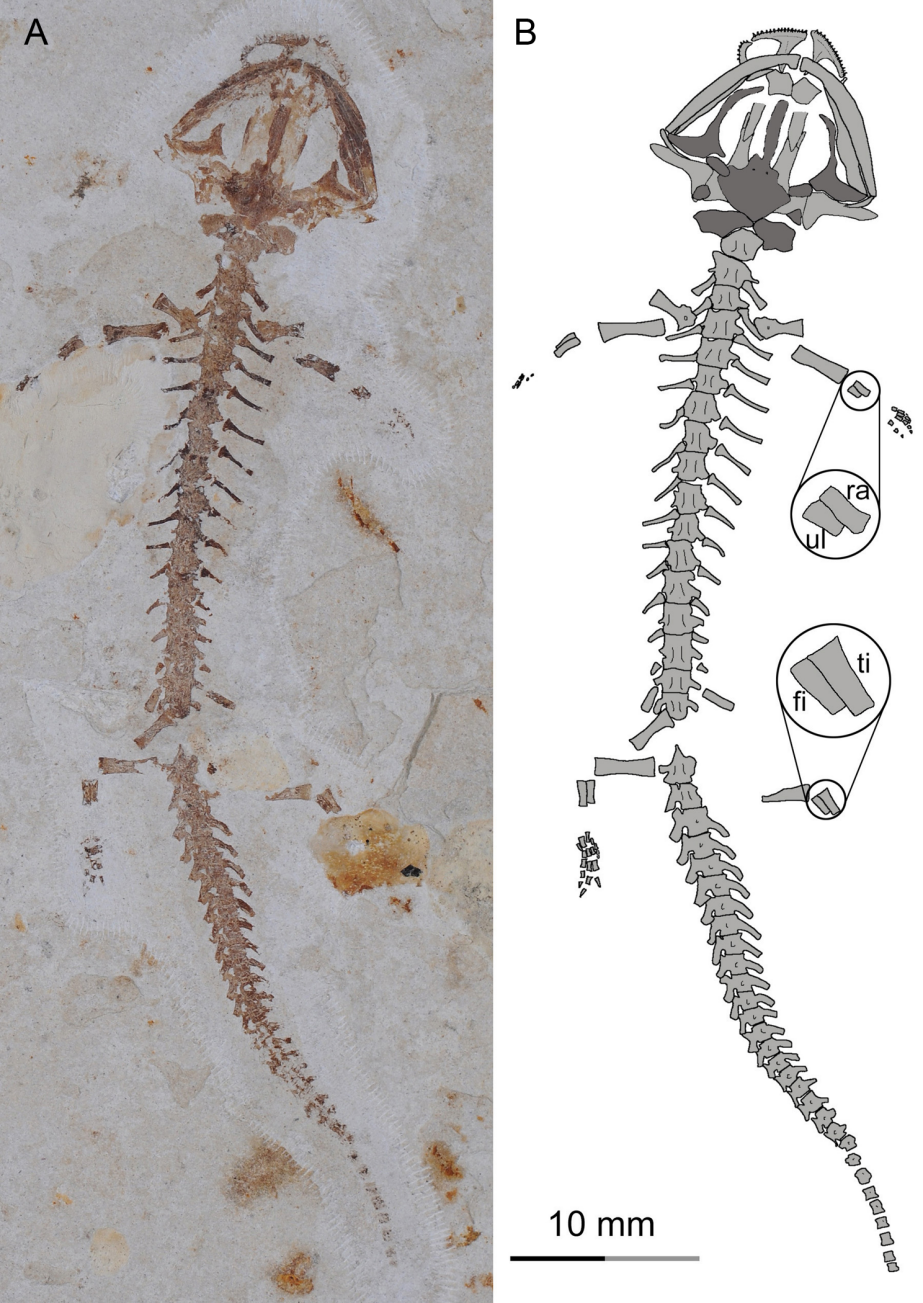
A larval specimen of *Nuominerpeton aquilonaris* gen. et sp. nov. (PKUP V0420): photograph (A) and line drawing (B) of the articulated skeleton in ventral view. Note the premetamorphic shape of the palatopterygoid, and longer radius than ulna in the arm and longer tibia than fibula in the leg (magnified insets in line drawing) indicating preaxial polarity in limb development. Dark shades denote palatal and braincase elements.

The humerus is straight with a short shaft and expanded proximal and distal ends. The epiphyses are fully ossified at both ends in adult specimens, but not in larval and juvenile specimens. Also in adult specimens, the crista dorsalis (dorsal crest) develops on the extensor side of the proximal end of the humerus as a robust ridge with a pronounced tubercle (PKUP V0414) for attachment of the M. subscapularis (Francis, 1934). The crista ventralis (ventral crest) arises as a large and roughly triangular process from the flexor side, serving for attachment of the M. pectoralis and M. supra-coracoideus (Francis, 1934). At the distal end, the humerus has a large radial condyle and a relatively small ulnar condyle. The trochlear groove is a shallow sulcus between the two condyles.

The radius is straight with strongly expanded proximal and distal ends, and a short shaft constricted midway along the bone. In contrast, the ulna is slightly curved, and is obviously wider proximally than distally. The radius is slightly shorter than the ulna. It distally has an oblique surface for articulation with the radiale, intermedium and the centrale 2. The ulna distally articulates with the intermedium and ulnare (Figs. 2, 5 and 8). In the smallest larval specimen (PKUP V0420; Fig. 9), the radius is longer and stouter than the ulna, indicating preaxial polarity in limb development as seen in extant salamanders (Shubin & Wake, 1996; Shubin & Wake, 2003), as recently reported in the temnospondyl taxa *Micromelerpeton* and *Sclerocephalus* (Fröbisch et al., 2015) and previously in the branchiosaurid *Apateon* (Fröbisch, Carroll & Schoch, 2007).

Carpal elements are well ossified in adults, but not in larval and juvenile specimens. A total of nine elements are identified in the extensively ossified limb in PKUP V0415 and V0421 (Figs. 5 and 8), comprising three basal elements (radiale, ulnare, intermedium), two centralia and a series of distal elements (basale commune, distal carpals 3 and 4, element y). In the holotype, all of these carpal elements are ossified, except for the radiale, indicating that it is a slightly younger individual than PKUP V0415 and V0421. As seen in the latter specimens, the radiale is significantly smaller than the ulnare. Centrale 2 articulates with the radius and separates the radiale from the intermedium as seen in some species of *Batrachuperus*, *Liua shihi*, *Ranodon*, *Paradactylodon* (Deinega, 1917; Zhang, 1985; Shubin & Wake, 2003; AmphibiaTree, 2004), but it does not separate the radiale from the intermedium as in *Salamandrella* and *Cryptobranchus* (Reese, 1906; Ma, 1964).

**Figure 10:**
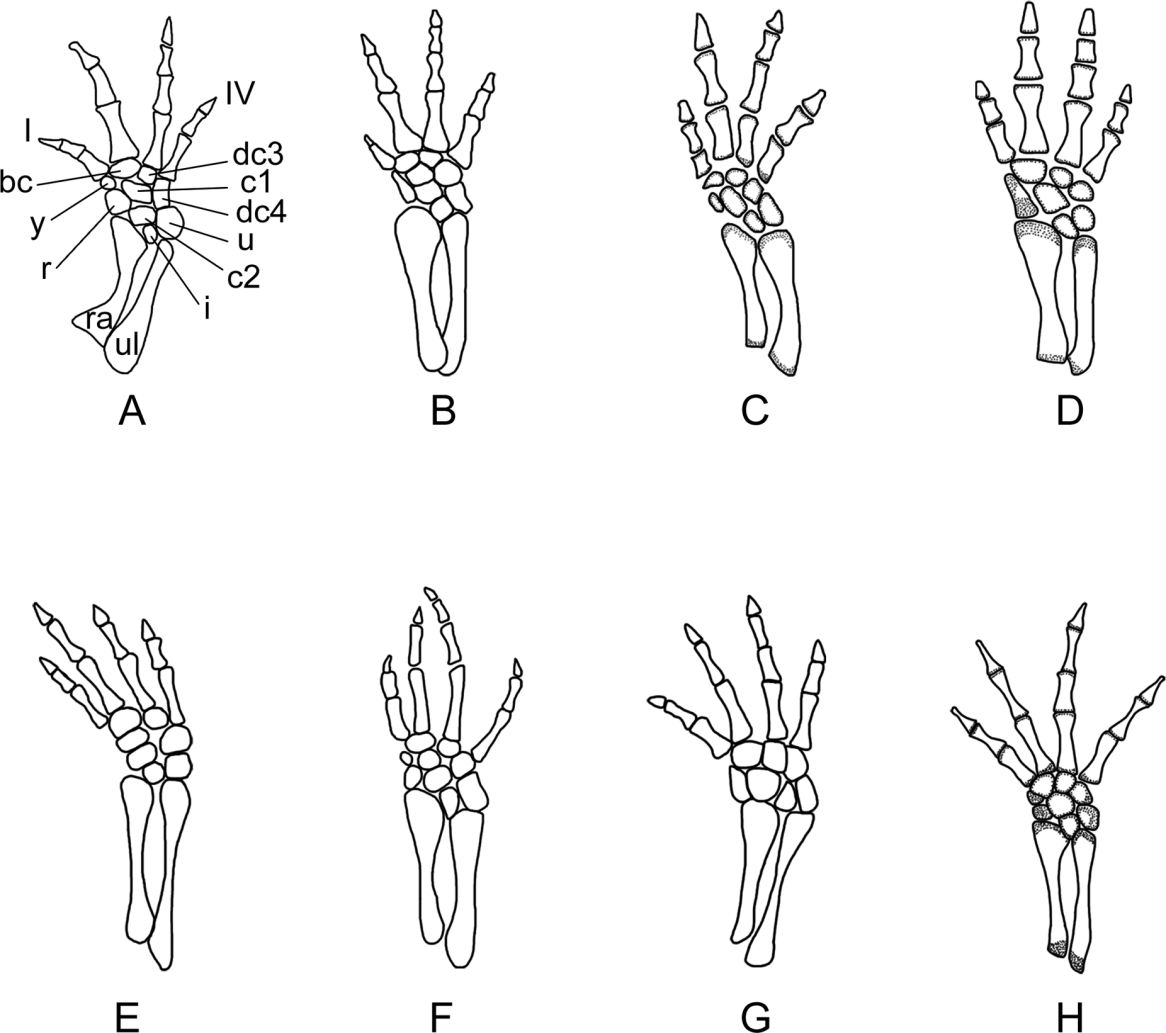
Right forelimb of the new salamander (*Nuominerpeton aquilonaris* gen. et sp. nov.) in comparison with patterns in extant hynobiids. (A) *Nuominerpeton*, reconstruction based on PKUP V0415; (B) *Ranodon sibiricus*; (C) *Hynobius leechii*; (D) *Batrachuperus pinchonii*; (E) *Salamandrella keyserlingii*; (F) *Liua shihi*; (G) *Pachyhynobius shangchengensis*; (H) *Onychodactylus* sp. Dotted area indicates cartilage. Not to scale.

Adult specimens display the basale commune, an amalgamation of distal carpals 1 and 2, which articulates with metacarpal I and II as seen in extant salamanders (Shubin & Wake, 1996; Shubin & Wake, 2003). As the smallest bone in the carpus, element y articulates with the radiale and centrale 1 proximally, and with the basale commune and metacarpal I distally (Fig. 10).

**Figure 11:**
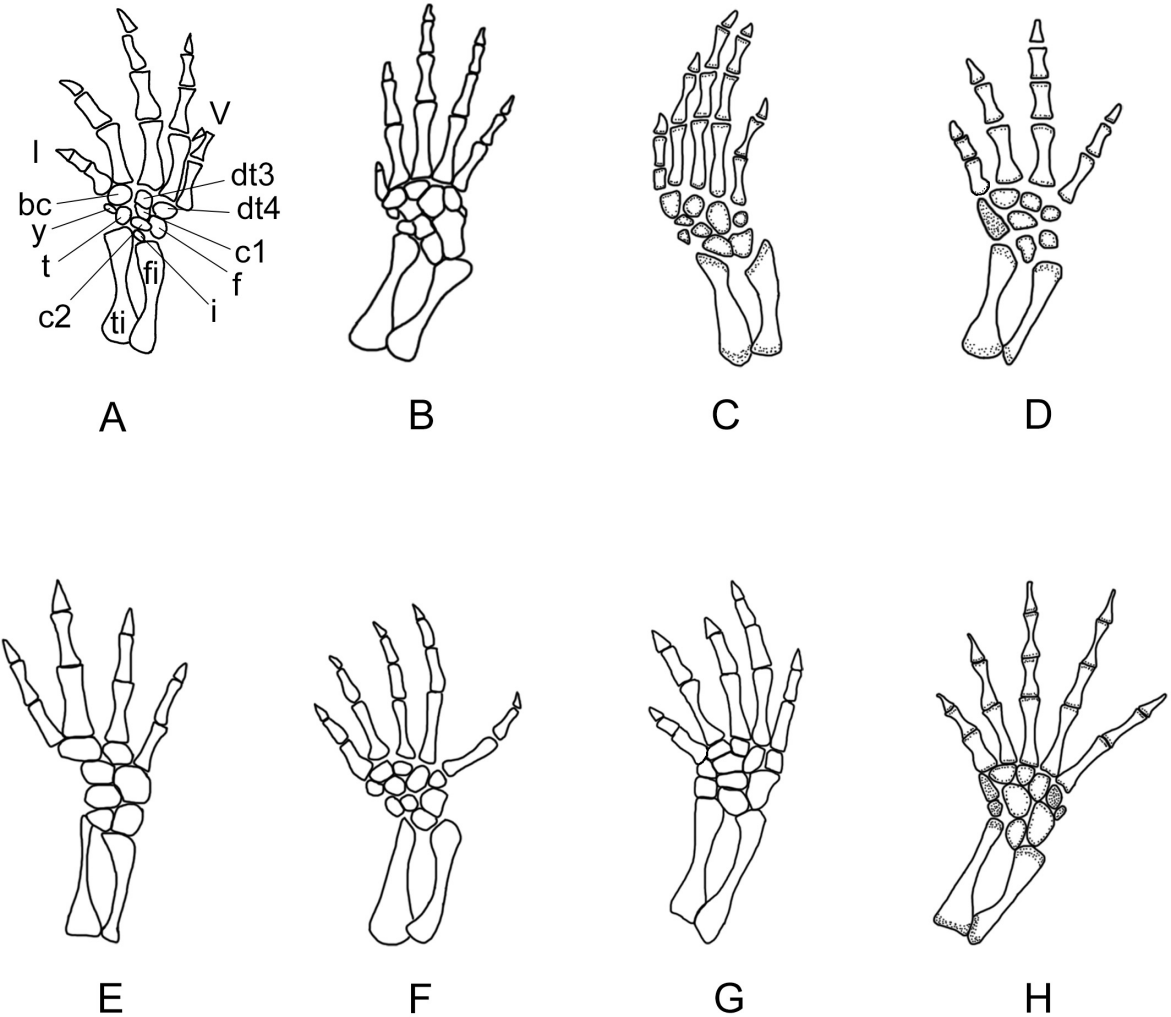
Right hind limb of the new salamander (*Nuominerpeton aquilonaris* gen. et sp. nov.) in comparison with patterns in extant hynobiids. (A) *Nuominerpeton aquilonaris*, reconstruction based on PKUP V0415; (B) *Ranodon sibiricus*; (C) *Hynobius leechii*; (D) *Batrachuperus pinchonii*; (E) *Salamandrella keyserlingii*; (F) *Liua shihi*; (G) *Pachyhynobius shangchengensis*; (H) *Onychodactylus* sp. Dotted area indicates cartilage. All in dorsal view and not to scale.

Metacarpal I is the shortest, with its proximal end strongly expanded. Metacarpal II is the longest and is expanded to be the stoutest among the metacarpals. Metacarpal III is slightly longer than IV. The phalangeal formula is 2-2-3-2, a common pattern for salamanders (Shubin & Wake, 1996; Shubin & Wake, 2003). Digit 3 is the longest, whereas digit 2 is longer than digit 4. As consistently observed in all specimens, digit 1 is the shortest, being merely half the length of digit 2. A comparable condition is seen in *Hynobius leechii* and *Batrachuperus* among extant hynobiids (Figs. 10C and 10D). Among other hynobiids, an extremely short digit 1 occurs in *Ranodon* (Deinega, 1917; Fig. 10B), whereas in the remaining hynobiid taxa digit 1 is more than half the length of digit 2 (Ma, 1964; Ma & Ma, 1987; Zhang, 1985; Zhao & Zhang, 1985; Wang, Zhao & Liang, 2004; Figs. 11E–11H). Based on current hypotheses for the phylogenetic relationships within the hynobiid clade (Zhang et al., 2006; Chen et al., 2015), a short digit 1 is apparently a derived feature acquired independently in *Ranodon*, *Hynobius* and *Batrachuperus*. The new salamander described in this paper adds to the distribution of this derived feature by extending its occurrence (presumably independently) to a fossil form of Early Cretaceous age.

The ossified pelvic girdle consists of paired ilia and ischia, whereas the cartilaginous pubis and ypsiloid remain unossified as commonly seen in extant salamanders (Duellman & Trueb, 1986). As observed in several specimens (PKUP V0414, V0415, V0422), the ilium is roughly club-shaped, with its expanded ventral plate investing part of the acetabulum, and a dorsal ‘head’ slightly wider than the shaft. The acetabulum is a shallow concavity, on the posteroventral part of the ilium in combination with the ischium. The ischium is best shown in PKUP V0421 as a kidney-shaped plate with a concave dorsal border. The plate is anteriorly widened with a straight edge, but posteriorly narrow with a rounded border. The paired plates meet ventrally to form the symphysis.

The femur is slightly curved, and roughly the same length as the humerus. The epiphyses of the femur are well ossified at both the proximal and distal ends in adults, but not in larval specimens (PKUP V0419, V0420). The condition remains unclear in the juvenile specimen (PKUP V0416) because the femur is poorly preserved. On the flexor side, the trochanter projects as a prominent and twig-like process, which is set well apart from the proximal end, but close to the shaft (Figs. 3, 5 and 8). The trochanteric crest (femoral crest) extends from the trochanter to the mid-level of the shaft. The trochanteric groove is short and shallow along the trochanteric crest. The distal end of the femur is expanded to form a large tibial condyle and a small fibular condyle. The trochlear groove is deep, separating the two condyles on the flexor side at the distal end of the femur.

Both the tibia and fibula are straight, and are roughly equal in length; but the former element is more robust than the latter. The tibia is wider proximally than distally, whereas the fibula conversely is wider distally than proximally. The tibia articulates with the intermedium distally, whereas the fibula articulates with the fibulare and intermedium. In the smallest larval specimen (PKUP V0420), the tibia is stouter and longer than fibula, reflecting the preaxial polarity in limb development as seen in extant salamanders (Shubin & Wake, 1996; Shubin & Wake, 2003), and in the temnospondyls *Micromelerpeton* and *Sclerocephalus* (Fröbisch et al., 2015) and the branchiosaurid *Apateon* (Fröbisch, Carroll & Schoch, 2007).

Adult specimens have as many as nine elements ossified in the tarsus, with distal tarsal 5 missing (Figs. 5, 8 and 11). Basal elements include a fibulare, an intermedium, and a tibiale. Two centralia are plesiomorphically present as ossified elements as in many extant hynobiids (Shubin & Wake, 2003; see Fig. 11). The Early Cretaceous *Liaoxitriton zhongjiani* also displays such a plesiomorphic pattern (Shubin & Wake, 2003). Distal elements include a basale commune, distal tarsal 3 and 4, and element y. The basale commune represents the amalgamation of distal tarsals 1 + 2, similar to the above-described amalgamation of distal carpals 1 + 2 in the carpus (Shubin & Wake, 1996; Shubin & Wake, 2003). Distal tarsal 4 has a narrow proximal end entering the central region of the tarsus, and is larger than the basale commune and distal tarsal 3 as seen in extant hynobiids (Fig. 11). Distal tarsal 5 probably remained cartilaginous, based on the vacant space consistently present between metatarsal V and the fibulare. Distal tarsal 4 and 5 are separate in all extant cryptobranchoids, except some variants of *Cryptobranchus* and the four-toed *Salamandrella*, in which the two elements are fused to form a large amalgamation, i.e., distal tarsal 4 + 5 (Shubin & Wake, 2003; Fig. 11E). In the new salamander, element y articulates with metatarsal I, the basal commune, centrale 1 and the tibiale.

Metatarsal III is the longest and metatarsal I the shortest, with the former being roughly twice the length of the latter. Metatarsal IV is longer than II, which in turn is longer than metatarsal V. Metatarsal I is strongly expanded at its proximal end as in many extant hynobiids, including *Liua* (Zhao & Zhang, 1985), *Pachyhynobius* (Zhao & Zhang, 1985), *Ranodon* (Deinega, 1917) and *Batrachuperus* (Zhang, Liu & Zhao, 2009). All phalanges are similar in shape to the metatarsals except for the terminal ones being subtriangular in shape. The phalangeal formula is 2-2-3-3-2, a plesiomorphic pattern in salamanders (Shubin & Wake, 2003). Digit 3 is the longest and digit 1 the shortest. Digit 4 is longer than digit 2, which in turn is longer than digit 1. Similar to the manus, digit 1 is reduced to about half the length of digit 2. A similar condition is seen in *Hynobius*, *Batrachuperus* and *Pachyhynobius* (Zhao & Zhang, 1985; Ma & Ma, 1987; Zhang, Liu & Zhao, 2009; Figs. 11C, 11D and 11G). *Ranodon* displays a strongly reduced digit 1, being even shorter than metatarsal II (Deinega, 1917; Fig. 11B). Other hynobiids have the first toe obviously longer than half-length of the second toe (Ma, 1964; Zhang, 1985; Wang, Zhao & Liang, 2004; Figs. 10E, 10F and 10H).

## Results and Discussion

As has been described above, the fossil material used in this study includes larval, juvenile and adult specimens. Comparative study of these specimens provides a rare opportunity for understanding developmental features in a fossil salamander of Early Cretaceous age. This study revealed several significant developmental features, among which the following are worthy of discussion:

### Types of larvae and gill rakers

Among extant salamanders, three adaptive types of larvae are recognized based on their ecological adaptations (Valentine & Dennis, 1964): pond-type larvae have a pair of rod-like balancers, long gill filaments, and deep and thin caudal fins; stream-type larvae tend to have short but bushy gill filaments, low and fleshy caudal fins, but lack balancers; and mountain brook-type larvae have extremely short and stubby gill rami, no gill rakers or present remnantly, no balancers, and reduced caudal fins limited to the distal half of the tail (Valentine & Dennis, 1964; Worthington & Wake, 1971; Duellman & Trueb, 1986; Lauder & Shaffer, 1993).

The four larval specimens (PKUP V0417–V0420) used in this study were possibly of the mountain brook-type, because none of them show gill rakers. The absence of external gills may be the result of those soft-tissue structures not being preserved. Alternatively, the lack of any trace of gill rakers may well reflect the actual absence of those structures as occurs in mountain brook-type larvae of extant salamanders. However, because all available specimens are relatively large larvae with extensive ossification of the skull and postcranium, possible early resorption of gill structures before approaching the onset of metamorphosis cannot be ruled out. Impressions of caudal fins are not preserved in these larval specimens, although reduced caudal fins are expected. All specimens of the new salamander are preserved in volcanic shales deposited in a pond environment. We interpret that the larval, juvenile and adult salamanders lived in a near-pond mountain-brook environment, like the extant hynobiids *Onychodactylus*, *Liua* and some species of *Batrachuperus* (Fei et al., 2006); but soon after death were carried by mountain-brook current into the pond, and fossilized in shale deposits in the pond. A recent study has identified both pond-type and stream-type larvae in the fossil record from the Jurassic of northern China (Gao, Chen & Jia, 2013). If correctly interpreted, the discovery of mountain brook-type larvae here documents all three adaptive larval types of salamanders in the Mesozoic fossil record from China and indicates those larval types appeared by at least the Early Cretaceous.

### Resorption of palatine and remodeling of palatopterygoid

As a significant life-history feature in metamorphic salamanders, metamorphosis marks the transformation from larval to juvenile and eventually to the adult stage (Rose & Reiss, 1993). Along with this life-history transition comes the dramatic remodeling of the palatopterygoid bone, coupled with lateral rotation of the palatal process of the pterygoid (Rose, 2003; Lebedkina, 2004). As a common pattern in metamorphic salamanders, a palatopterygoid first develops with the palatine portion contacting the pterygoid portion, whereas the elongate anterior process of the palatopterygoid curves anteromedially. The palatine and anterior pterygoid portions of the palatopterygoid start to be resorbed prior to metamorphosis, and both completely vanish at metamorphosis (Worthington & Wake, 1971; Smirnov & Vassilieva, 2002a; Rose, 2003). Accompanying this resorption process, the palatal process of the pterygoid turns anterolaterally to transform into a postmetamorphic type of pterygoid. As discussed below, these changes also occur in the new salamander taxon described in this study.

In the four larval specimens (PKUP V0417–V0420), the palatine portion of the palatopterygoid is present with variable extents. Three specimens (PKUP V0418–V0420) with SPLs ranging between 33.9 mm–43.8 mm have the palatine portion slightly expanded and bearing multiple rows of teeth. The palatine portion is continuous without interruption with the pterygoid process of the palatopterygoid (Figs. 7A, 7B and 9). Another specimen (PKUP V0417: Fig. 7C) represents a larva obviously larger than the others judging from the size of the skull, but with unknown SPL length. In this specimen the palatine and anterior pterygoid portions of the palatopterygoid are resorbed substantially, and only an extremely thin thread without teeth remains (Fig. 7C). This specimen is interpreted as having died as it approached metamorphosis, a rare find in the salamander fossil record.

PKUP V0416 (SPL of 47 mm) is a postmetamorphic juvenile. It has the palatine and anterior portions of the palatopterygoid completely resorbed, and the shortened pterygoid process is re-oriented anterolaterally (Figs. 6 and 7D). Its unossified mesopodium (Fig. 6) indicates that this individual died shortly after metamorphosis, before adulthood. Because this postmetamorphic juvenile has a skull similar in size to that of the large larval form (PKUP V0417), we hypothesize that the thread-like anterior portion of the palatopterygoid in the latter specimen reflects resorption of the palatopterygoid without breaking of the palatine portion. Such a resorption pattern has been documented in the hynobiids *Onychodactylus*, *Ranodon* and *Salamandrella* (Lebedkina, 1964; Smirnov & Vassilieva, 2002a; Vassilieva, Poyarkov & Iizuka, 2013).

### Orbitosphenoid and nerve pathways

Most salamanders have an ossified orbitosphenoid in form of a bony plate contributing to the side wall of the braincase, but exceptions are known for proteids (Gilbert, 1973; Rose, 2003), some species of the plethodontid *Eurycea* (Wake, 1966; DigiMorph Staff, 2003), and the recently reported basal salamandroid *Qinglongtriton* of Late Jurassic age (Jia & Gao, 2016). Previous studies have shown that patterns of nerve foramina in association with the orbitosphenoid in salamanders are phylogenetically informative, but little attention has been paid to this character in previous phylogenetic analyses (Duellman & Trueb, 1986; Zhao et al., 1988; Trueb & Cloutier, 1991; Larson & Dimmick, 1993; Gao & Shubin, 2001; Gao & Shubin, 2012).

Among salamanders with an ossified orbitosphenoid, four different patterns can be recognized in terms of the relative positions of the optic and oculomotor nerve foramina: (1) The optic foramen penetrates the orbitosphenoid, with the oculomotor foramen opening in a cartilaginous plate posterior to the orbitosphenoid. This pattern is seen in cryptobranchids (Reese, 1906; Qiu & Yang, 1986) and some species of plethodontids (e.g., *Ensatina*; Wake, 1963); (2) The optic foramen opens at the notched posterior edge of the orbitosphenoid, with the oculomotor foramen opening posteriorly in a large gap covered by a cartilaginous plate. Such a pattern is seen in hynobiids, ambystomatids and rhyacotritonids (see description above; AmphibiaTree, 2007a; AmphibiaTree, 2007b); (3) The optic foramen penetrates the orbitosphenoid, with the oculomotor foramen opening at the posterior edge of the orbitosphenoid. This pattern occurs in some species of plethodontids (e.g., *Karsenia*), some salamandrids (e.g., *Salamandra*), dicamptodontids, and amphiumids (Wake, 1963; Carroll & Holmes, 1980; Buckley, Wake & Wake, 2010; Wu, Wang & Hanken, 2012; Villa et al., 2014); (4) Both the optic and oculomotor foramina penetrate the orbitosphenoid as seen in some species of plethodontids (e.g., *Karsenia*), some salamandrids (e.g., *Pachytriton*), and sirenids (Reilly & Altig, 1996; Wake, 1963; Wu, Wang & Hanken, 2012). No information is available for the optic and oculomotor foramina in any of the known stem caudates (e.g., *Karaurus*, *Kokartus*, *Marmorerpeton*, *Urupia*), leaving the four patterns outlined above unpolarized.

Extant hynobiids uniformly have the posterior margin of the orbitosphenoid notched for the optic nerve, with the oculomotor foramen penetrating the cartilaginous plate posterior to the orbitosphenoid (Deinega, 1917; Ma, 1964; Zhao & Zhang, 1985; Rose, 2003; Fei et al., 2006; Zhang, 1985; Zhang, Liu & Zhao, 2009). The same pattern seen in extant hynobiids also occurs in the new salamander described in this paper and also in *Liaoxitriton zhongjiani* (pers. obs. of specimens in PKUP collection by both authors), the latter of which has been recognized as a basal hynobiid (Chen & Gao, 2009). In contrast, *Pangerpeton* displays a different pattern: the optic foramen penetrates the orbitosphenoid, with the oculomotor foramen opening at the notched posterior margin of the same element (PKUP V0222). This pattern is in line with the above-mentioned pattern 3, but differs from patterns in extant hynobiids and cryptobranchids (i.e., conditions 2 and 1, respectively). Clearly, the significance of the positions of the optic and oculomotor foramina relative to the orbitosphenoid as a character in the phylogeny of urodeles needs to be tested in a cladistic analysis (work in progress).

### Ossification of hyobranchial elements

Salamanders display complex ossification patterns of the hyobranchial apparatus, and variable patterns in the hyobranchium involving loss and fusion of elements in different taxonomic groups (Trueb, 1993; Rose, 2003). As a general pattern for the two unpaired median elements in all salamanders, basibranchial I is present either as a cartilaginous or ossified element, whereas basibranchial II can be ossified with variable shapes or simply absent (Trueb, 1993; Rose, 2003; Jia & Gao, 2016). In association with the median elements are the hypohyal and ceratohyal, hypobranchials and ceratobranchials. Many of these hypobranchial elements remain cartilaginous, with hypobranchials and some ceratobranchials often ossified variably in different taxonomic groups.

As described above, the new salamander *Nuominerpeton* has an unossified basibranchial I as in all hynobiids except *Onychodactylus*, in which the element is partly ossified as a short stub (Smirnov & Vassilieva, 2002a; Xiong et al., 2013a). In *Nuominerpeton* basibranchial II is ossified as an anchor-shaped structure, a plesiomorphic pattern in urodeles (Jia & Gao, 2016), but not seen in any extant hynobiids (Xiong et al., 2013a). Interestingly, the ossified basibranchial II is only seen in adult specimens, not in larval or postmetamorphic juvenile specimens. This suggests that ossification of the basibranchial II is a postmetamorphic phenomenon in this fossil salamander. Because ossification sequence of the basibranchial is rarely documented in the literature, a direct comparison of this fossil salamander with extant hynobiids in this aspect is impossible.

Also in the new salamander, hypobranchial I and ceratobranchial I remain unossified, a general pattern seen in all hynobiids except *Pachyhynobius*, in which the former element is ossified although the latter element remains cartilaginous (Xiong et al., 2013a). Like in all hynobiids, both hypobranchial II and ceratobranchial II in the new salamander are well ossified as rod-like structures in adults. Comparison of specimens used in this study in different developmental stages indicates that hypobranchial II is ossified early before metamorphosis, whereas ceratobranchial II seems to be ossified at metamorphosis. This interpretation is supported by our observation that all available postmetamorphic specimens of the new salamander display an ossified hypobranchial II and ceratobranchial II, whereas only hypobranchial II is present in larval specimens. Such an ossification sequence is similar to that reported for *Onychodactylus fischeri* (Smirnov & Vassilieva, 2002a) and *Onychodactylus japonicus* (Vassilieva, Poyarkov & Iizuka, 2013), but differs from *Hynobius formosanus*, in which both hypobranchial II and ceratobranchial II do not ossify until after metamorphosis (Vassilieva et al., 2015).

By comparison with other crown-group salamanders, it appears that ossification of hypobranchial II and ceratobranchial II as separate elements is characteristic for Cryptobranchoidea, with an independent occurrence in *Proteus* but not in *Necturus*; in the latter proteid both elements remain cartilaginous (Gilbert, 1973). More variable ossification patterns of these elements in adults can be briefly outlined in other salamander groups as follows: hypobranchial II is ossified and ceratobranchial II remains cartilaginous in sirenids (Reilly & Altig, 1996); hypobranchial II is absent and ceratobranchial II is cartilaginous in *Amphiuma* (Erdman & Cundall, 1984); and hypobranchial II remains cartilaginous and ceratobranchial II is absent in ambystomatids, salamandrids, plethodontids, rhyacotritonids and dicamptodontids (Wake, 1966; Özeti & Wake, 1969; Worthington & Wake, 1971; Krogh & Tanner, 1972).

**Figure 12:**
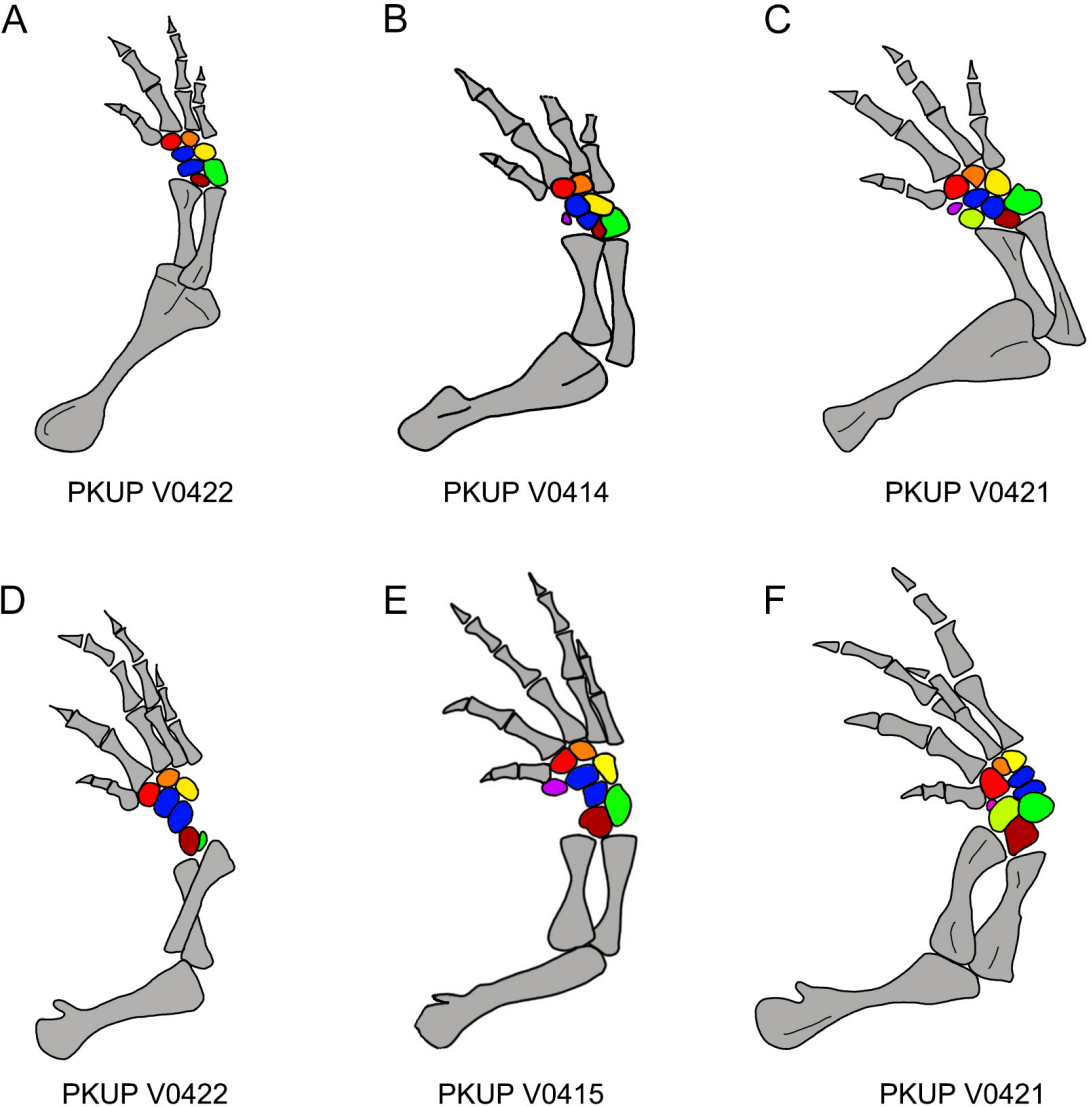
Ossification sequence of mesopodial elements (color coded) in the new salamander (*Nuominerpeton aquilonaris* gen. et sp. nov.). (A–C) Right fore limb; (D–F) right hindlimb. Note that element y is ossified before radiale in manus and before tibiale in pes. Color codification: blue-centralia; purple-element y; red-basale commune; orange-distal carpal/tarsal 3; yellow-distal carpal/tarsal 4; green-ulnare/fibulare; maroon-intermedium; lime-radiale/tibiale.

### Limb ossification

In salamanders, ossification of the mesopodium is delayed until after metamorphosis or it remains cartilaginous throughout life (Shubin & Wake, 1996; Shubin & Wake, 2003). Thus, because larval salamander fossils are rare, patterns of mesopodial ossification are rarely documented in the fossil record. Among Mesozoic urodeles, mesopodial elements are completely unossified in all neotenic and some metamorphic salamanders (e.g., *Valdotriton* and *Pangerpeton*), whereas those elements are incompletely ossified in other metamorphic salamanders (*Laccotriton*, *Liaoxitriton*, *Sinerpeton*, *Regalerpeton* and *Iridotriton*). The new fossil taxon described herein documents the most extensive ossification sequence of the mesopodium for any known Mesozoic salamander.

With ossification of nine elements in both the carpus and tarsus, the mesopodium of the new salamander displays a plesiomorphic pattern in having two centralia in the manus and pes (Fig. 12). Among extant cryptobranchoids, two centralia occur as ossified elements in the hynobiids *Liua*, *Ranodon*, *Salamandrella*, *Paradactylodon*, *Pachyhynobius* and some species of *Batrachuperus* (Deinega, 1917; Ma, 1964; Zhao & Zhang, 1985; Shubin & Wake, 2003). Two centralia are also found as cartilaginous elements in the cryptobranchid *Cryptobranchus* (Shubin & Wake, 2003). Among other cryptobranchoids, a single centrale is found ossified in the hynobiids *Hynobius* and *Onychodactylus*, and present as cartilaginous in the cryptobranchid *Andrias* (Shubin & Wake, 2003). The actual condition remains unknown in the hynobiids *Pseudohynobius* and *Protohynobius*.

Among the Mesozoic salamanders outlined above, two centralia are known for the manus in *Laccotriton* (Gao & Shubin, 2001), whereas only one centrale occurs in the manus of *Sinerpeton* (Gao & Shubin, 2001). *Liaoxitriton zhongjiani* was figured with two centralia in the pes (Dong & Wang, 1998; Shubin & Wake, 2003); our observation of referred specimens of the same taxon in Peking University collections confirms two centralia also occur in the manus. The Late Jurassic *Iridotriton* has a well-ossified carpus and tarsus, with the carpus displaying fusion of the ulnare and intermedium (Evans et al., 2005); however, the actual number of centrale elements remains unknown. The Early Cretaceous *Regalerpeton weichangensis* is known by a single specimen (IVPP V14391), which has one possible carpal and six tarsal elements preserved as impressions on the right fore- and hind limbs (Zhang et al., 2009), but the identity of these mesopodial elements, and thus, the number of centralia remains unclear. Nonetheless, all these fossil taxa with ossified mesopodium are members of Cryptobranchoidea, based on the presence of unicapitate ribs (Gao & Shubin, 2012; Gao, Chen & Jia, 2013), with *Liaoxitriton zhongjiani* recognized as a stem hynobiid (Chen & Gao, 2009).

In addition to documenting a rare fossil example of an extensively ossified mesopodium, the new hynobiid-like salamander also provides evidence on the ossification sequences of mesopodial elements in the carpus and tarsus. Among the available specimens, a small adult (PKUP V0422: SPL of ∼70 mm) has most elements ossified except element y and the radiale/tibiale (Figs. 12A and 12D). The holotype (PKUP V0414: SPL of 77.7 mm) has most elements ossified except the radiale in the carpus (tarsus uncertain). PKUP V0415 (SPL of ∼78 mm) has a fully ossified carpus, but retaining an unossified tibiale in the tarsus (Figs. 12B and 12E). The largest adult known (PKUP V0421: SPL of 79.8 mm) has both a fully ossified carpus and tarsus (Figs. 12C and 12F). This comparison of adult specimens of different sizes indicates a distal-to-proximal ossification sequence for the preaxial column (radiale/tibiale and element y) in both the carpus and tarsus; that is, element y ossifies earlier than the radiale/tibiale. Such an ossification sequence of the preaxial column is difficult to compare with extant salamanders, because ossification sequences in the carpus and tarsus are poorly documented for extant salamanders (Fröbisch, 2008). PKUP V0415 also shows the carpus completes its ossification before the tarsus. In that specimen, the tibiale remains unossified in the tarsus whereas in the carpus the corresponding radiale is fully ossified. This delayed ossification of the preaxial column suggests that the digital arch (basale commune and distal carpals), central column (intermedium and centralia) and postaxial column (ulnare and distal carpals) ossify earlier than the preaxial column. The mesopodium as a whole in the new fossil taxon achieved its full ossification later in adulthood, perhaps after reaching sexual maturity as occurs in extant salamanders (Fröbisch, 2008). Extensive ossification of the mesopodium in both the carpus and tarsus indicates that this new salamander was well adapted for terrestrial locomotion.

### Phylogenetic relationships, paleobiogeography and paleoclimate

The new salamander can be confidently classified in Cryptobranchoidea, on the basis of two features: retention of an angular in the lower jaw and possession of unicapitate ribs (Duellman & Trueb, 1986; Fei et al., 2006). In addition, this study provides evidence from an Early Cretaceous fossil taxon confirming shared derived features by all members of the cryptobranchoid clade: ossification of both hypobranchial II and ceratobranchial II as separate elements in the hyobranchium, with hypobranchial I and ceratobranchial I remaining cartilaginous.

Within Cryptobranchoidea, the new salamander appears to be more closely affiliated with Hynobiidae than with Cryptobranchidae. The new taxon lacks any derived features seen in cryptobranchids, including the loss of the lacrimal, anterior extension of the frontal separating the nasal from the maxilla, and the closure of the anteromedial fenestra in the palate. In contrast, both the presence of a lacrimal bone and the optic foramen opening at the notched posterior border of the orbitosphenoid suggest that the new salamander is more closely related to hynobiids.

Our descriptive study of the new salamander taxon has identified a suite of features suggesting a close relationship with hynobiids. This hypothesized relationship will be tested as part of a large scale phylogenetic analysis of salamanders currently in progress by us. If the relationship is supported, the new salamander from Inner Mongolia will add to the Early Cretaceous record of the family and will lend support to the hypothesis based on molecular phylogeny that Hynobiidae originated roughly 120–150 Ma in Asia (Zhang et al., 2006; Chen et al., 2015).

As mentioned above, several hynobiid-like salamanders (*Liaoxitriton*, *Laccotriton*, *Sinerpeton*, *Reglerpeton*) have been found from the Lower Cretaceous beds within the Jehol area in northern China. Our new find is made from a locality at least 450 km north to the Jehol area, close to the 49th parallel north. No Cretaceous hynobiid-like salamanders worldwide, by our knowledge, have reached such a northern distribution, whereas the *Salamandrella* and *Ranodon* fossils from Russia as mentioned in our introduction are Miocene and Pliocene in age, respectively; thus, at least 100 Ma younger than our Early Cretaceous fossil record. Our new discovery also implies that northbound dispersal of hynobiid-like salamanders from the Jehol area might have taken place during Early Creteceous Aptian time (∼122 Ma) in East Asia.

Previous studies have shown that the Jehol area has already set at the current mid-latitude position roughly between 40–45th parallel north (Enkin et al., 1992; Yang et al., 2013). The Jehol area probably had an mean annual air temperature of 10 ± 4 °C during the Early Cretaceous Barremian–early Albian interval, as estimated based on oxygen isotope composition of apatite phosphate (*δ*^18^O_p_) from the remains of various group of reptiles recovered from the Jehol beds (Amiot et al., 2011). Such a climate condition is in accordance with the temperature range that most extant hynobiid species tolerate (Fei et al., 2006: 6.5–24 °C), except that *Salamandrella* can survive extremely cold weather conditions (AmphibiaWeb, 2016). Because our new hynobiid-like salamander is found from a higher latitude (48.6°N) than the Jehol area, an even cooler air temperature is expected at this latitude in comparison to the Jehol area.

## Conclusions

The following conclusions can be drawn from our study of the new salamander taxon from the Lower Cretaceous Guanghua Formation of Inner Mongolia:

(1) *Nuominerpeton aquilonaris* (gen. nov. sp. nov.) is a hynobiid-like salamander characterized by having a semicircular-shaped orbitosphenoid, a strongly expanded coracoid portion of the scapulocoracoid with a convex ventral and posterior borders, and substantially shortened first digit in both the manus and pes.
(2) Comparative study of the four adult, one juvenile and four larval specimens reveals developmental changes in the cranial and postcranial skeleton, specifically: increasing ossification of the orbitosphenoid, articular, and mesopodial elements; resorption of the coronoid in the lower jaw, palatine and anterior portions of the palatopterygoid; ceratobranchial II ossifying later than hypobranchial II; and a distoproximal ossification sequence for preaxial mesopodial elements in the manus and pes.
(3) The new fossils provide a rare example of ossification sequence in the limb skeleton for a fossil salamander. In this Early Cretaceous taxon, the preaxial column of the mesopodium ossifies later than the digital arch, central column and postaxial column; and the carpus completely ossifies before the tarsus.
(4) The discovery of *Nuominerpeton aquilonaris* documents the first Mesozoic salamander in China from outside the Jehol area, extending the geographic distribution of Mesozoic salamanders in China from 40th–45th to far north close to the 49th parallel north. Thus, the new discovery also documentes the northernmost geographic distribution of hynobiid-like salamanders in the Cretaceous world.
(5) Phylogenetically, the new salamander shares with hynobiids two derived features: optic foramen opens at the notched posterior border of the orbitosphenoid and hypobranchial II and ceratobranchial II ossify as separate elements, whereas hypobranchial I and ceratobranchial I remain cartilaginous. The new discovery adds to the previously reported fossil record of hynobiid-like salamanders from the Cretaceous in China, providing further support to the hypothesis derived from molecular data that the Hynobiidae originated during the Early Cretaceous in East Asia.

## Institutional abbreviations

HGM: Hungarian Geological Museum, Budapest, Hungary
IVPP: Institute of Vertebrate Paleontology and Paleoanthropology, Chinese Academy of Sciences, Beijing, China
MTC: Țării Crișurilor Museum, Oradea, Romania
PKUP: Peking University Paleontological Collections, Beijing, China
ZIN: Zoological Institute, Russian Academy of Sciences, St. Petersburg, Russia

## Anatomical abbreviations

adf: anterodorsal fenestra
amf: anteromedial fenestra
an: angular
ar: articular
at: atlas
bb: basibranchial
bc: basale commune
c: centrale
cb: ceratobranchial
co: coronoid
crd: crista dorsalis
crv: crista ventralis
d: dentary
dc: distal carpal
dt: distal tarsal
f: fibulare
fe: femur
fi: fibula
fr: frontal
hb: hypobranchial
hu: humerus
i: intermedium
icaf: internal carotid foramen
il: ilium
isc: ischium
lac: lacrimal
m: maxilla
na: nasal
obs: orbitosphenoid
opi-exo: opisthotic-exoccipital complex
pa: parietal
pm: premaxilla
ppt: palatopterygoid
pra: prearticular
prf: prefrontal
pro: prootic
ps: parasphenoid
pt: pterygoid
qu: quadrate
r: radiale
ra: radius
sca: scapulocoracoid
sm: septomaxilla
sq: squamosal
st: stapes
t: tibiale
ti: tibia
u: ulnare
ul: ulna
vo: vomer
y: element y

## Additional Information and Declarations

### Data Availability

The following information was supplied regarding data availability: No raw data is involved in this research.

### New Species Registration

The following information was supplied regarding the registration of a newly described species: *Nuominerpeton aquilonaris* gen. et sp. nov. LSID urn:lsid:zoobank.org:act:2BCD03D7-3D14-47B2-8C20-63FE0F59B958; Genus *Nuominerpeton* gen. nov. LSID: zoobank.org:act:1D7FC3AE-4E45-4FBE-8504-E6C9DA12369E; Publication LSID: urn:lsid:zoobank.org:pub:B71B1033-62FB-4183-802A-34C431C9CC3D.

## References

[ref-1] Amiot R, Wang X, Zhou Z, Wang X, Buffetaut E, Lécuyer C, Ding Z, Fluteau F, Hibino T, Kusuhashi N, Mo J, Suteethorn V, Wang Y, Xu X, Zhang F (2011). Oxygen isotopes of East Asian dinosaurs reveal exceptionally cold Early Cretaceous climates. Proceedings of the National Academy of Sciences of the United States of America.

[ref-2] AmphibiaTree (2004). “*Batrachuperus persicus*”. Digital Morphology. http://digimorph.org/specimens/Batrachuperus_persicus/.

[ref-3] AmphibiaTree (2007a). “*Onychodactylus japonicus*”. Digital Morphology. http://digimorph.org/specimens/Onychodactylus_japonicus/head/.

[ref-4] AmphibiaTree (2007b). “*Rhyacotriton variegatus*”. Digital Morphology. http://digimorph.org/specimens/Rhyacotriton_variegatus/head/.

[ref-5] AmphibiaWeb (2016). Information on amphibian biology and conservation.

[ref-6] Averianov AO, Tjutkova LA (1995). *Ranodon cf. sibiricus* (Amphibia, Caudata) from the Upper Pliocene of southern Kazakhstan: the first fossil record of the family Hynobiidae. Paläontologische Zeischrift.

[ref-7] Buckley D, Wake MH, Wake DB (2010). Comparative skull osteology of *Karsenia koreana* (Amphibia, Caudata, Plethodontidae). Journal of Morphology.

[ref-8] Carroll RL, Holmes R (1980). The skull and jaw musculature as guides to the ancestry of salamanders. Zoological Journal of the Linnean Society.

[ref-9] Chang S, Zhang H, Hemming SR, Mesko GT, Fang Y, Jourdan F, Mark DF, Verati C (2014). ^40^Ar/^39^Ar age constraints on the Haifanggou and Lanqi formations: when did the first flowers bloom?. Advances in ^40^Ar/^39^Ar dating: from archaeology to planetary sciences.

[ref-10] Chang S, Zhang H, Renne PR, Fang Y (2009). High-precision 40Ar/39Ar age constraints on the basal Lanqi Formation and its implications for the origin of angiosperm plants. Earth and Planetary Science Letters.

[ref-11] Chen J, Gao KQ (2009). Early Cretaceous hynobiid *Liaoxitriton zhongjiani* (Amphibia: Caudata) from Liaoning, China, and the monophyly of the Hynobiidae. Journal of Vertebrate Paleontology.

[ref-12] Chen MY, Mao RL, Liang D, Kuro-o M, Zeng XM, Zhang P (2015). A reinvestigation of phylogeny and divergence times of Hynobiidae (Amphibia, Caudata) based on 29 nuclear genes. Molecular Phylogenetics and Evolution.

[ref-13] Clemen G, Greven H (2009). Sex dimorphic dentition and notes on the skull and hyobranchium in the hynobiid salamander *Pachyhynobius shangchengensis* Fei, Qu & Wu, 1983 (Urodela: Amphibia). Vertebrate Zoology.

[ref-14] Deinega VA (1917). To the knowledge on structure and development of *Ranodon sibiricus* Kessel. Bulletin de La Société Imperiale des Naturalistes de Moscou, Annee 1916, N. S..

[ref-15] DigiMorph Staff (2003). “*Eurycea robusta*”. Digital Morphology. http://digimorph.org/specimens/Eurycea_robusta/head/.

[ref-16] Dong ZM, Wang Y (1998). A new urodele (*Liaoxitriton zhongjiani* gen. et sp. nov.) from the Early Cretaceous of Western Liaoning Province, China. Vertebrata PalAsiatica.

[ref-17] Duellman WE, Trueb L (1986). Biology of amphibians.

[ref-18] Duméril AMC (1806). Zoologie analytique, ou méthode naturelle de classification des animaux, rendue plus facile à l’aide de tableaux synoptiques.

[ref-19] Dunn ER (1922). The sound-transmitting apparatus of salamanders and the phylogeny of the Caudata. American Naturalist.

[ref-20] Dunn ER (1923). The salamanders of the Family Hynobiidae. Proceedings of the American Academy of Arts and Sciences.

[ref-21] Edwards JL (1976). Spinal nerves and their bearing on salamander phylogeny. Journal of Morphology.

[ref-22] Elwood JRL, Cundall D (1994). Morphology and behavior of the feeding apparatus in *Cryptobranchus alleganiensis* (Amphibia: Caudata). Journal of Morphology.

[ref-23] Enkin RJ, Yang Z, Chen Y, Courtillot V (1992). Paleomagnetic constraints on the geodynamic history of the major blocks of China from the Permian to the Present. Journal of Geophysical Research.

[ref-24] Erdman S, Cundall D (1984). The feeding apparatus of the salamander *Amphiuma tridactylum*: morphology and behavior. Journal of Morphology.

[ref-25] Estes R (1981). Encyclopedia of paleoherpetology, Part 2: Gymnophiona, Caudata.

[ref-26] Evans SE, Lally C, Chure DC, Elder A, Maisano JA (2005). A Late Jurassic salamander (Amphibia: Caudata) from the Morrison Formation of North America. Zoological Journal of the Linnean Society.

[ref-27] Fei L, Hu S, Ye C, Huang Y (2006). Fauna sinica amphibia Volume 1.

[ref-28] Fei L, Ye CY (2000). A new hynobiid subfamily with a new genus and new species of Hynobiidae from west China. Cultum Herpetologica Sinica.

[ref-29] Fox H (1959). A study of the development of the head and pharynx of the larval urodele *Hynobius* and its bearing on the evolution of the vertebrate head. Philosophical Transactions of the Royal Society of London. Series B: Biological Sciences.

[ref-30] Francis ETB (1934). The anatomy of the Salamander.

[ref-31] Fröbisch NB (2008). Ossification patterns in the tetrapod limb-conservation and divergence from morphogenetic events. Biological Reviews.

[ref-32] Fröbisch NB, Bickelmann C, Olori JC, Witzmann F (2015). Deep-time evolution of regeneration and preaxial polarity in tetrapod limb development. Nature.

[ref-33] Fröbisch NB, Carroll RL, Schoch RR (2007). Limb ossification in the Paleozoic branchiosaurid *Apateon* (Temnospondyli) and the early evolution of preaxial dominance in tetrapod limb development. Evolution and Development.

[ref-34] Frost DR (2016). http://research.amnh.org/herpetology/amphibia/index.html.

[ref-35] Gao KQ, Chen JY A new crown-group frog (Amphibia: Anura) from the Lower Cretaceous of northeastern Inner Mongolia, China. American Museum Novitates.

[ref-36] Gao KQ, Chen J, Jia J (2013). Taxonomic diversity, stratigraphic range, and exceptional preservation of Juro-Cretaceous salamanders from northern China. Canadian Journal of Earth Sciences.

[ref-37] Gao KQ, Cheng ZW, Xu X (1998). First report of Mesozoic urodeles from China. Chinese Geology.

[ref-38] Gao KQ, Shubin NH (2001). Late Jurassic salamanders from northern China. Nature.

[ref-39] Gao KQ, Shubin NH (2003). Earliest known crown-group salamanders. Nature.

[ref-40] Gao KQ, Shubin HN (2012). Late Jurassic salamandroid from western Liaoning, China. Proceedings of the National Academy of Sciences of the United States of America.

[ref-41] Gilbert SG (1973). Pictorial anatomy of the Necturus.

[ref-42] Goodrich ES (1930). Studies on the structure and development of vertebrates.

[ref-43] Greven H, Clemen G (1985). Morphological studies on the mouth cavity of Urodela VIII. The teeth of the upper jaw and the palate in two *Hynobius*-species (Hynobiidae: Amphibia). Sonderdruck aus Zeitschrift für zoologische Systematik und Evolutionsforschung.

[ref-44] Haeckel E (1866). Generelle morphologie der organismen.

[ref-45] Hecht MK, Edwards JL, Hecht MK, Goody PC, Hecht BM (1977). The methodology of phylogenetic inference above the species level. Major patterns in vertebrate evolution.

[ref-46] Heilongjiang Institute of Geological Survey (2005). Reports on regional geology of the People’s Republic of China (Arun section).

[ref-47] Ivachnenko M (1978). Urodeles from the Triassic and Jurassic of Soviet Central Asia. Paleontological Journal.

[ref-48] Jia J, Gao KQ (2016). A new basal salamandroid (Amphibia, Urodela) from the Late Jurassic of Qinglong, Hebei Province, China. PLoS ONE.

[ref-49] Joubert PJ (1961). Contributions to the cranial morphology of *Pseudotritonruber ruber* (Sonnini). Annale Universiteit Van Stellenbosch.

[ref-50] Khozatski LI, Shantser EV, Nikiforova KV (1982). Amphibians. Stratigraphy of the USSR. Quaternary system 1.

[ref-51] Krogh JE, Tanner WW (1972). The hyobranchium and throat myology of the adult Ambystomatidae of the United States and northern Mexico. Brigham Young University Science Bulletin.

[ref-52] Larson A, Dimmick WW (1993). Phylogenetic relationships of the salamander families: an analysis of congruence among morphological and molecular characters. Herpetological Monographs.

[ref-53] Lauder GV, Shaffer HB, Hanken J, Hall BK (1993). Design of feeding systems in aquatic vertebrates: Major patterns and their evolutionary interpretations. The skull, 3: functional and evolutionary mechanisms.

[ref-54] Lebedkina NS (1964). The development of the dermal bones of the basement of the skull in Urodela (Hynobiidae). Trudy Akademiia Nauk SSSR.

[ref-55] Lebedkina NS (2004). Evolution of the amphibian skull.

[ref-56] Linnaeus C (1758). Systema Naturae: per regna tria naturae, secundum classes, ordines, genera, species, cum characteribus, differentiis, synonymis, locis 1.

[ref-57] Liu T, Tong H, Li G, Xiang S (2008). Characteristics of lithofacies and depositional model of Mesozoic Dayangshu Basin. Journal of Daqing Petroleum Institute.

[ref-58] Ma KQ (1964). The skeletal system of *Hynobius keyserlingii*. Journal of Jilin Normal University.

[ref-59] Ma LD, Ma DK (1987). Morphology and osteology of *Hynobius leechii* (Amphibia: Hynobiidae). Chinese Journal of Zoology.

[ref-60] Meszoely C (1966). North American fossil cryptobranchid salamanders. American Midland Naturalist.

[ref-61] Nambu H (1991). *Hynobius tenuis* (Caudata, Hynobiidae), a new species of salamander from central Japan. Zoological Science.

[ref-62] Noble GK (1931). The biology of the amphibia.

[ref-63] Özeti N, Wake DB (1969). The morphology and evolution of the tongue and associated structures in salamanders and newts (Family Salamandridae). Copeia.

[ref-64] Peng R, Zhang P, Xiong JL, Gu HJ, Zeng XM, Zou FD (2010). Rediscovery of *Protohynobius puxiongensis* (Caudata: Hynobiidae) and its phylogenetic position based on complete mitochondrial genomes. Molecular Phylogenetics and Evolution.

[ref-65] Pyron RA, Wiens JJ (2011). A large-scale phylogeny of Amphibia including over 2,800 species, and a revised classification of extant frogs, salamanders, and caecilians. Molecular Phylogenetics and Evolution.

[ref-66] Qiu YX, Yang AF (1986). The osteological research of the Chinese giant salamander, *Megalobatrachus davidianus*. Acta Scientiarum Naturalium Universitatis Pekinensis.

[ref-67] Reese AM (1906). Anatomy of *Cryptobranchus alleghaniensis*. The American Naturalist.

[ref-68] Regal PJ (1966). Feeding specializations and the classification of terrestrial salamanders. Evolution.

[ref-69] Regel ED (1970). Ascending process of the palatoquadratic cartilage in urodelans. Doklady Akademii Nauk SSSR.

[ref-70] Reilly SM (1983). The biology of the high altitude salamander *Batrachuperus mustersi* from Afghanistan. Journal of Herpetology.

[ref-71] Reilly SM, Altig R (1996). Cranial ontogeny in *Siren intermedia*(Caudata: Sirenidae): paedomorphic, metamorphic, and novel patterns of heterochrony. Copeia.

[ref-72] Rose CS, Heatwole H, Davies M (2003). The developmental morphology of salamander skulls. Amphibian biology. Volume 5: osteology.

[ref-73] Rose CS, Reiss JO, Hanken J, Hall BK (1993). Metamorphosis and the vertebrate skull: ontogenetic patterns and developmental mechanisms. The skull. Volume 1: development.

[ref-74] Sato I (1943). A monograph of the tailed Batrachians of Japan.

[ref-75] Schmalhausen II (1958). Nasolacrimal duct and septomaxillare of Urodela. Zoologicheskii Zhurnal.

[ref-76] Schmalhausen II (1968). The origin of terrestrial vertebrates.

[ref-77] Scopoli JA (1777). Introductio ad historiam naturalem, sistens genera lapidum, plantarum et animalium hactenus detecta, caracteribus essentialibus donata, in tribus divisa, subinde ad leges naturae.

[ref-78] Sessions SK (2008). Evolutionary cytogenetics in salamanders. Chromosome Research.

[ref-79] Shubin NH, Wake DB (1996). Phylogeny, variation, and morphological integration. American Zoologist.

[ref-80] Shubin NH, Wake DB, Heatwole H, Davies M (2003). Morphological variation, development, and evolution of the limb skeleton of salamanders. Amphibian biology. Volume 5: osteology.

[ref-81] Skutschas PP (2013). Mesozoic salamanders and albanerpetontids of Middle Asia, Kazakhstan, and Siberia. Palaeobiodiversity and Palaeoenvironments.

[ref-82] Skutschas PP (2016). A new crown-group salamander from the Middle Jurassic of Western Siberia, Russia. Palaeobiodiversity and Palaeoenvironments.

[ref-83] Skutschas PP, Krasnolutskii SA (2011). A new genus and species of basal salamanders from the Middle Jurassic of western Siberia, Russia. Proceedings of the Zoological Institute RAS.

[ref-84] Skutschas PP, Martin T (2011). Cranial anatomy of the stem salamander *Kokartus honorarius* (Amphibia: Caudata) from the Middle Jurassic of Kyrgyzstan. Zoological Journal of the Linnean Society.

[ref-85] Smirnov SV, Vassilieva AB (2002a). Skeletal and dental ontogeny in the long-tailed clawed salamander, *Onychodactylus fischeri* (Urodela: Hynobiidae). Russian Journal of Herpetology.

[ref-86] Smirnov SV, Vassilieva AB (2002b). The bony skull of the Siberian salamander *Salamandrella keyserlingii* (Amphibia: Urodela: Hynobiidae) and the role of thyroid hormones in its development. Doklady Biological Sciences.

[ref-87] Sun G, Zhao H, Han Y, Ma J, Liu X, Sha H, Zhou S, Ma L (2005). Establishment of the Late Cretaceous Gushanzhen Formation in Arun Qi, eastern Inner Mongolia, China. Geological Bulletin of China.

[ref-88] Suzuki T (1932). Development of the brain, nervous system, and cranial skeleton in amphibians. I. Development of the skull in *Onychodactylus japonicus*. Kaibogaku Zassi (Acta Anatomica Japonica).

[ref-89] Syromyatnikova EV (2014). The first record of *Salamandrella* (Caudata: Hynobiidae) from the Neogene of Russia. Russian Journal of Herpetology.

[ref-90] Tjutkova LA (1990). Late Pliocene lagomorphs and rodents of southeastern Kazakhstan. D. Phil. Thesis.

[ref-91] Trueb L, Hanken J, Hall BK (1993). Patterns of cranial diversity among the Lissamphibia. The skull, volume 2: patterns of structural and systematic diversity.

[ref-92] Trueb L, Cloutier R, Schultze HP, Trueb L (1991). A phylogenetic investigation into the inter- and intrarelationships of the Lissamphibia (Amphibia: Temnospondyli). Origins of the higher groups of tetrapods: controversy and consensus.

[ref-93] Valentine BD, Dennis DM (1964). A comparison of the gill-arch system and fins of three genera of larval salamanders, *Rhyacotriton*, *Gyrinophilus*, and *Ambystoma*. Copeia.

[ref-94] Vasilyan D, Böhme M (2012). Pronounced peramorphosis in lissamphibians–*Aviturus exsecratus* (Urodela, Cryptobranchidea) from the Paleocene-Eocene thermal maximum of Mongolia. PLoS ONE.

[ref-95] Vasilyan D, Böhme M, Chkhikvadze VM, Semenov YA, Joyce WG (2013). A new giant salamander (Urodela, Pancryptobrancha) from the Miocene of Eastern Europe (Grytsiv, Ukraine). Journal of Vertebrate Paleontology.

[ref-96] Vassilieva AB, Lai JS, Yang SF, Chang YH, Poyarkov Jr NA (2015). Development of the bony skeleton in the Taiwan salamander, *Hynobius formosanus* Maki, 1922 (Caudata: Hynobiidae): heterochronies and reductions. Vertebrate Zoology.

[ref-97] Vassilieva AB, Poyarkov NA, Iizuka K (2013). Pecularities of bony skeleton development in asian clawed salamanders (*Onychodactylus*, Hynobiidae) related to embryonization. Biology Bulletin.

[ref-98] Vassilieva AB, Smirnov SV (2001). Development and morphology of the dentition in the Asian salamander, *Ranodon sibiricus* (Urodela: Hynobiidae). Russian Journal of Herpetology.

[ref-99] Venczel M (1999a). Land salamanders of the family Hynobiidae from the Neogene and Quaternary of Europe. Amphibia-Reptilia.

[ref-100] Venczel M (1999b). Fossil land salamanders (Caudata, Hynobiidae) from the Carpathian Basin: relation between extinct and extant genera. Acta Palaeontologica Romaniae.

[ref-101] Villa A, Andreone F, Boistel R, Delfino M, Capula M, Corti C (2014). Skull and lower jaw osteology of the Lanza’s salamander, *Salamandra lanzai* (Amphibia, Caudata). Scripta herpetologica: studies on amphibians and reptiles in Honour of Benedetto Lanza.

[ref-102] Wake DB (1963). Comparative osteology of the plethodontid salamander genus *Aneides*. Journal of Morphology.

[ref-103] Wake DB (1966). Comparative osteology and evolution of the lungless salamanders, family Plethodontidae. Memoir of the Southern California Academy of Sciences.

[ref-104] Wake DB, Lawson R (1973). Developmental and adult morphology of the vertebral column in the plethodontid salamander *Eurycea bislineata*, with comments on vertebral evolution in the Amphibia. Journal of Morphology.

[ref-105] Wang Y (2004). A new Mesozoic caudate (*Liaoxitriton daohugouensis* sp. nov.) from Inner Mongolia, China. Chinese Science Bulletin.

[ref-106] Wang L, Zhao Y, Liang C (2004). Studies on the skeletal system of *Onychodactylus fischeri* (Boulenger, 1886). Sichuan Journal of Zoology.

[ref-107] Weisrock DW, Harmon LJ, Larson A (2005). Resolving deep phylogenetic relationships in salamanders: analyses of mitochondrial and nuclear genomic data. Systematic Biology.

[ref-108] Weisrock DW, Macey JR, Matsui M, Mulcahy DG, Papenfuss TJ (2013). Molecular phylogenetic reconstruction of the endemic Asian salamander family Hynobiidae (Amphibia, Caudata). Zootaxa.

[ref-109] Wiens JJ, Bonett RM, Chippindale PT (2005). Ontogeny discombobulates phylogeny: paedomorphosis and higher-level salamander relationships. Systematic Biology.

[ref-110] Worthington RD, Wake DB (1971). Larval morphology and ontogeny of the ambystomatid salamander, *Rhyacotriton olympicus*. American Midland Naturalist.

[ref-111] Worthington RD, Wake DB (1972). Patterns of regional variation in the vertebral column of terrestrial salamanders. Journal of Morphology.

[ref-112] Wu YK, Wang YZ, Hanken J (2012). Comparative osteology of the genus *Pachytriton* (Caudata: Salamandridae) from southeastern China. Asian Herpetological Research.

[ref-113] Xiong JL, Gu HJ, Gong TJ, Zeng XM (2011). Redescription of an enigmatic salamander, *Pseudohynobius puxiongensis* (Fei & Ye, 2000) (Urodela: Hynobiidae). Zootaxa.

[ref-114] Xiong JL, Sun P, Zhang JL, Liu XY (2013a). A comparative study of the hyobranchial apparatus in Hynobiidae (Amphibia: Urodela). Zoology.

[ref-115] Xiong JL, Yu P, Zhang JL, Zhu WW, Sun P (2013b). Vertebral column characteristics of *Batrachuperus pinchonii*, and discussion on the division of the vertebral column in Urodela. Chinese Journal of Zoology.

[ref-116] Yang WB, Niu HC, Sun WD, Shan Q, Zheng YF, Li NB, Li CY, Arndt NT, Xu X, Jiang YH, Yu XY (2013). Isotopic evidence for continental ice sheet in mid-latitude region in the supergreenhouse Early Cretaceous. Scientific Reports.

[ref-117] Zhang FJ (1985). On anatomy of the skeletal system of *Liua shihi* (Liu) (Amphibia: Hynobiidae). Acta Herpetologica Sinica.

[ref-118] Zhang P, Chen YQ, Zhou H, Liu YF, Wang XL, Papenfuss TJ, Wake DB, Qu LH (2006). Phylogeny, evolution, and biogeography of Asiatic Salamanders (Hynobiidae). Proceedings of the National Academy of Sciences of the United States of America.

[ref-119] Zhang L, Gao K, Wang L (2004). New discovery of salamander fossils from the Yixian Formation in western Liaoning. Geological Bulletin of China.

[ref-120] Zhang H, Liu S, Zhao Y (2009). Skeletal system of *Batrachuperus pinchonii*. Sichuan Journal of Zoology.

[ref-121] Zhang G, Wang Y, Jones MEH, Evans SE (2009). A new Early Cretaceous salamander (*Regalerpeton weichangensis* gen. et sp. nov.) from the Huajiying Formation of northeastern China. Cretaceous Research.

[ref-122] Zhao EM, Hu QX, Jiang YM, Yang YH (1988). Studies on Chinese salamanders.

[ref-123] Zhao EM, Zhang FJ (1985). Comparative studies on the skeletons of *Ranodon*, *Batrachuperus*, *Liua* and *Xenobius* and their phylogeny. Acta Herpetologica Sinica.

